# Supplementary material for: Structural basis of the promiscuity of the unusual Fe(II) and 2-oxoglutarate dependent human aspartate/asparagine-β-hydroxylase
Source: Nat Commun. 2026 Feb 25;17:4267. doi: 10.1038/s41467-026-69425-w (PMC13168262; doi:10.1038/s41467-026-69425-w)
Supplement: Supplementary file 1 — Supplementary Information [file 41467_2026_69425_MOESM1_ESM.pdf]

Supplementary information

Structural basis of the promiscuity of the unusual Fe and 2-oxoglutarate dependent human aspartate/asparagine-β-hydroxylase

Mariska de Munnik[1,#], Amelia Brasnett[1,#], Tiankun Zhou[2,3], William Myers[4], Yicheng Wang[1], Kuntal Chatterjee[5,6], Anthony Tumber[1], Stephen Marshall[1], Philipp S. Simon[5], Pierre Aller[2,3], Anastasiia Shilova[2,3], Danny Axford[2], Hiroki Makita[5], Daniel W. Paley[5], Vandana Tiwari[5,7], Alexander T. Stead[1], Sebastian Dehe[7], Humberto Sanchez[7], Daniel J. Rosenberg[7], Roberto Alonso-Mori[7], Asmit Bhowmick[5], Junko Yano[5], Vittal K. Yachandra[5], Jaehyun Park[8], Sehan Park[8], Allen M. Orville[2], Lennart Brewitz[1,*], Jan F. Kern,[5,*] Christopher J. Schofield[1,*] and Patrick Rabe[1,2,*]

# These authors contributed equally to the work.

*E-mail: lennart.brewitz@chem.ox.ac.uk, jfkern@lbl.gov, christopher.schofield@chem.ox.ac.uk or patrick.rabe@chem.ox.ac.uk

Author affiliations

[1]Chemistry Research Laboratory and the Ineos Oxford Institute for Antimicrobial Research, University of Oxford, 12 Mansfield Road, OX1 3TA, Oxford, United Kingdom.

[2]Diamond Light Source, Diamond House, Harwell Science and Innovation Campus, Didcot OX11 0DE, United Kingdom.

[3] Research Complex at Harwell, Harwell Science and Innovation Campus, Didcot, Oxfordshire, OX11 0FA, United Kingdom.

[4]Centre for Advanced Electron Spin Resonance (CAESR), University of Oxford, South Parks Road, OX1 3QR, Oxford, United Kingdom.

[5]Molecular Biophysics and Integrated Bioimaging Division, Lawrence Berkeley National Laboratory, 1 Cyclotron Road, Berkeley, CA 94720, United States of America.

[6]Present address: Lam Research, Tualatin, OR 97062, United States of America.

[7]Linac Coherent Light Source, SLAC National Accelerator Laboratory, Menlo Park, CA 94025, United States of America.

[8]Pohang Accelerator Laboratory, Pohang University of Science and Technology, Pohang, 37673, Republic of Korea.

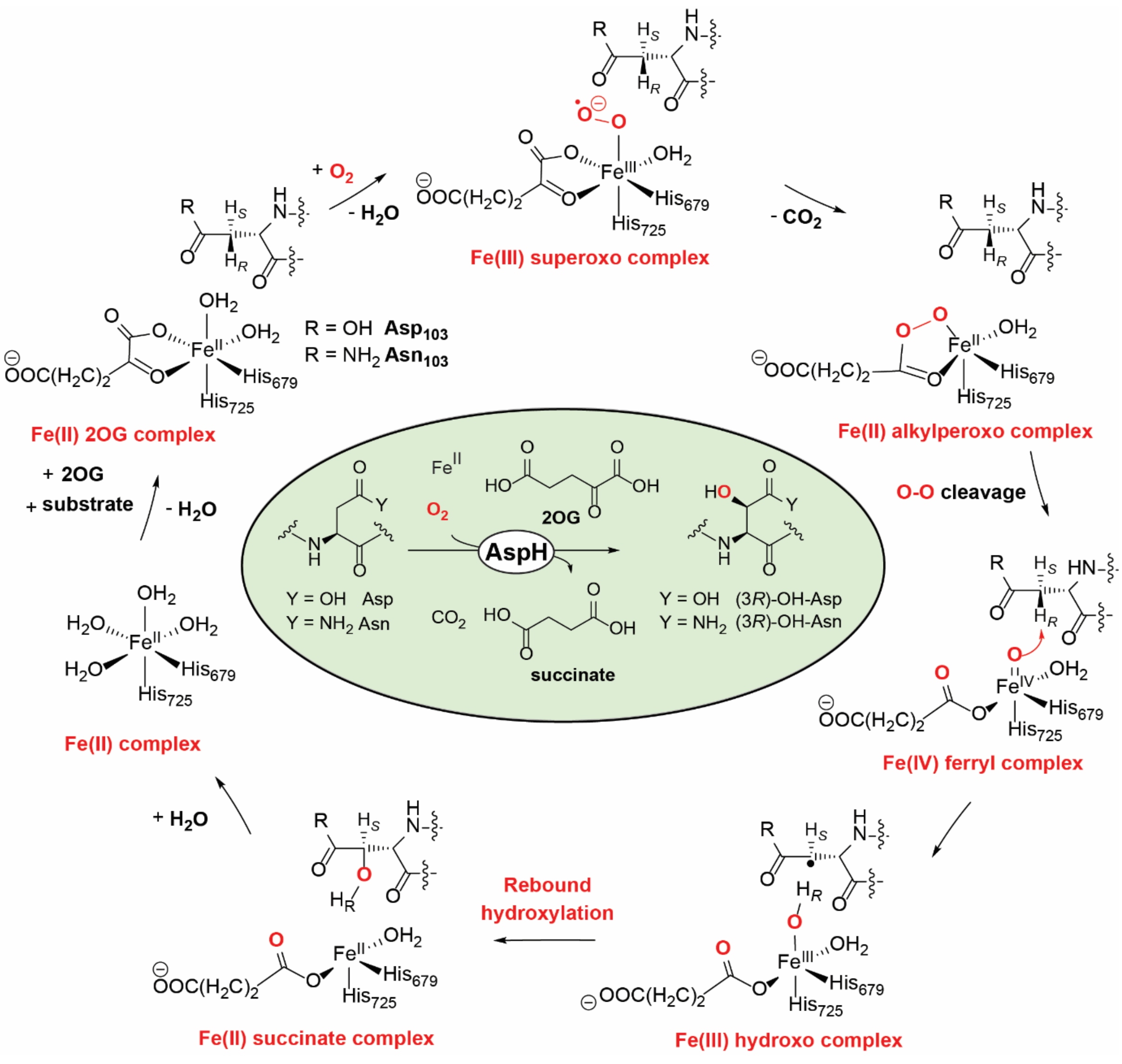

Supplementary Figure 1: Proposed outline mechanism for AspH catalysed hydroxylation. The reaction involves binding of the cosubstrate 2OG and hFX-EGFD1 forming an AspH:Fe(II):2OG:substrate complex, followed by O_2 binding *trans* to His_{725} to form an Fe(III) superoxo complex.[1] Reaction of the superoxo-ion with the 2OG C2 ketone carbonyl group enables oxidative 2OG decarboxylation leading to a proposed Fe(II) alkylperoxo complex; O-O bond cleavage results in an Fe(IV) succinate complex.[2-8] Abstraction of the *pro*-(*R*)-hydrogen by the ferryl oxygen results in formation of an Fe(III) hydroxy complex;[9] hydroxyl rebound results in substrate-radical hydroxylation and formation of an Fe(II) succinate complex.[10] Note the position *trans* to the 2OG C2 ketone oxygen is occupied by a water in the case of AspH, whereas it is more typically occupied by an Asp- or Glu-residue in other 2OG oxygenases.[10,11] The mechanism is based on that proposed for more typical 2OG oxygenases and is supported by computational studies.[12]

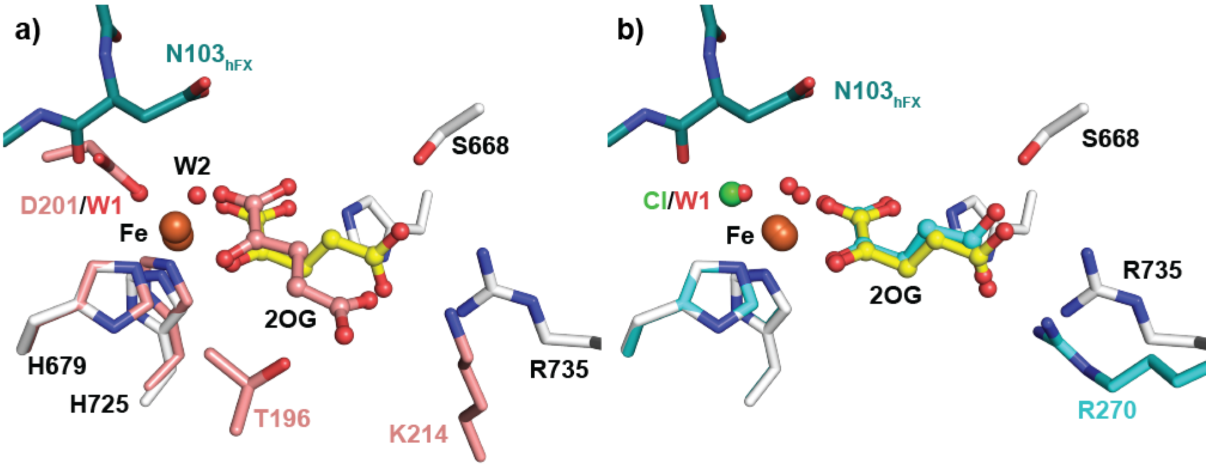

Supplementary Figure 2: Comparison of AspH active site with those of FIH and the 2OG dependent halogenase WelO5. a) Superimposition of AspH:Fe:2OG:hFX-Asp (PDB: 9FVZ, white; 2OG, yellow) with Factor Inhibiting HIF-1α (FIH):Fe:2OG (PDB: 1H2L, salmon) complex[13] revealing key differences in Fe-coordination, involving replacement of D201 in FIH with W1 in AspH. b) Superimposition of AspH:Fe:2OG:hFX-Asp (PDB: 9FVZ, white; 2OG, yellow) with the 2OG and Fe dependent halogenase WelO5:Fe:2OG:Cl (PDB: 5IQS, cyan) complex[14] revealing similar Fe-coordination, with W1 in AspH being replaced with a chloride ion in WelO5.

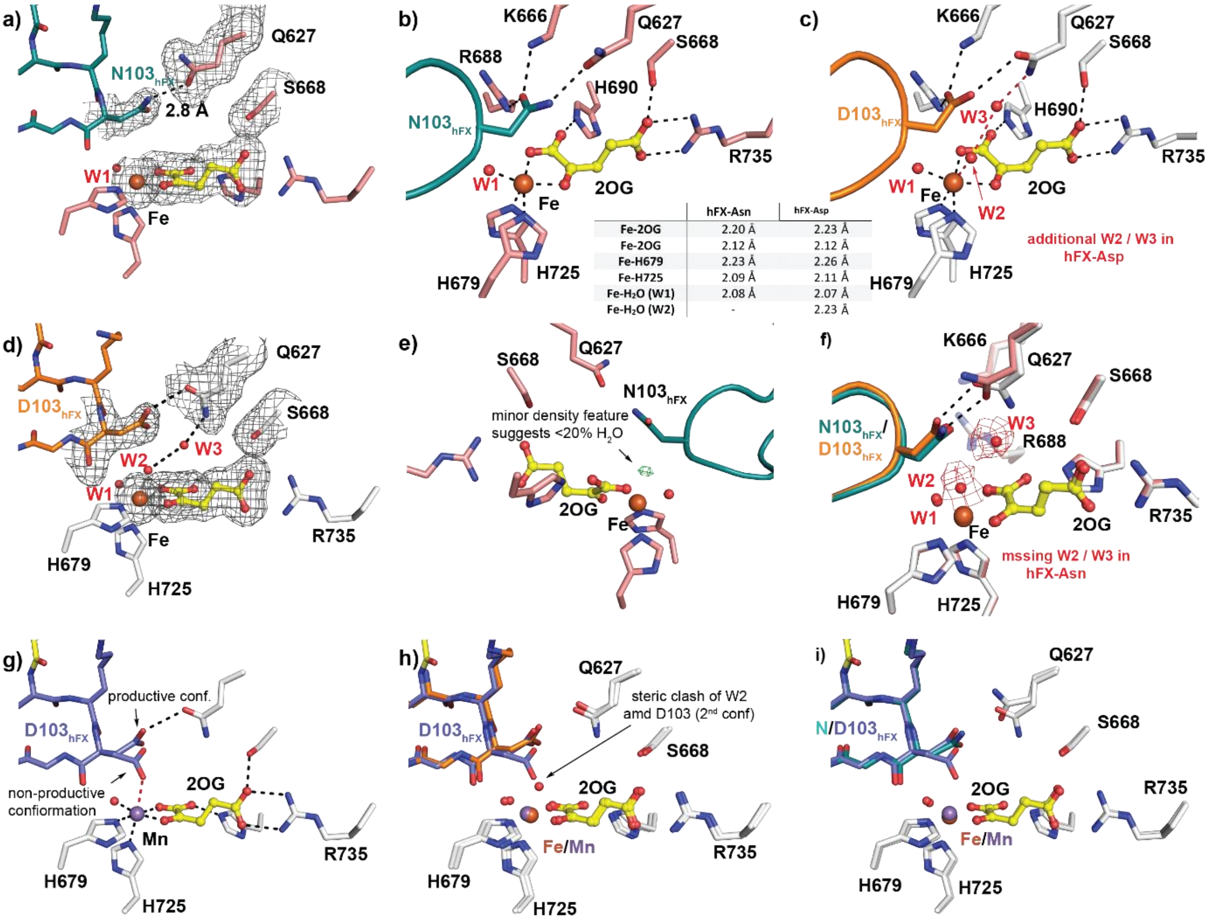

Supplementary Figure 3: Structures of the AspH:Fe/Mn:2OG complexes with hFX-Asp/Asn peptide fragments obtained under anaerobic conditions. a+d) Polder omit maps of: a) AspH:Fe:2OG:hFX-Asn (PDB: 9FVX, 1.90 Å resolution) and d) AspH:Fe:2OG:hFX-Asp (PDB: 9FVZ, 1.95 Å resolution) structures reveal similar conformations of active site residues, with the exception of an additional Fe-bound H_2O (W2) and W3 in the hFX-Asp structure. Note, the electron density maps indicate minor positive density in the AspH:Fe:2OG:hFX-Asn structure possibly reflecting low occupancy (~10 - 15%) binding of a H_2O molecule (see Supplementary Fig 3e). b+c) Active site coordination of substrate, Fe and 2OG of: b) AspH:Fe:2OG:hFX-Asn (PDB: 9FVX) and c) AspH:Fe:2OG:hFX-Asp (PDB: 9FVZ) reveal two additional H_2O molecules (W2 and W3) in the hFX-Asp structure compared to the hFX-Asn structure; additional hydrogen bonds: red dashed lines. Difference maps are shown in Supplementary Fig 3f. e) mF_o-DF_c maps of anaerobic AspH:Fe:2OG:hFX-Asn (PDB: 9FVX) revealing minor green positive electron density in the vacant Fe site likely due to partial H_2O binding <20%. f) Superimposition and $F_{obs} - F_{obs}$ isomorphous difference map of the active site of AspH:Fe:2OG:hFX-Asn (PDB: 9FVX) relative to AspH:Fe:2OG:hFX-Asp (PDB: 9FVZ, main chain RMSD: 0.152 Å) supporting the presence of two additional H_2O molecules in the hFX-Asp structure, implying a stronger interaction of Q627 with Asp103 compared to Asn103. g) Active site coordination of AspH:Mn:2OG:hFX-Asp (PDB: 8RE9)[15] implying the presence of the hFX-Asp side chain in two conformations: conformation 1) Q627-hydrogen bonded to the Asp103 side chain, corresponding to a catalytically productive conformation and conformation 2) Asp103 sidechain coordination to Mn, corresponding to a non-productive conformation, likely preventing O_2 binding. Superimposition of AspH:Mn:2OG:hFX-Asp (PDB: 8RE9)[15] relative to h) AspH:Fe:2OG:hFX-Asp (PDB: 9FVZ, main chain RMSD = 0.156 Å) and i) AspH:Fe:2OG:hFX-Asn (PDB: 9FVX, main chain RMSD: 0.157 Å) indicating the implications of Mn-bound non-productive conformation for Fe-bound W2 and potential O_2 binding.

Supplementary Figure 4: Direct competition studies imply hFX-Asp is a better AspH substrate than hFX-Asn. a-f) The apparent turnover numbers (k_{cat}^{app}) and Michaelis constants (K_m^{app}) of isolated recombinant $AspH_{315-758}$ were determined for 2OG and Fe(II), monitoring the AspH-catalysed hydroxylation of hFX-Asn (Figure 1) by solid phase extraction coupled to mass spectrometry (SPE-MS), as described.[16] Conditions: $AspH_{315-758}$ (0.05 µM), hFX-Asn (4.0 µM), L-ascorbic acid (LAA; 100 µM), and the shown concentrations of 2OG and $(NH_4)_2Fe(SO_4)_2{\cdot}6H_2O$ in buffer (25 mM HEPES, pH 7.5, 50 mM NaCl, 20 °C). Measurement times were normalized to the first sample injection analysed after addition of AspH to the Substrate Mixture (t = 0 s), by which time low levels of substrate oxidation were manifest. Data are means of independent triplicates (n = 3; mean ± SD).

a) Time course analysis of the AspH-catalysed oxidation of hFX-Asn (Figure 1) for the shown concentrations of 2OG using $(NH_4)_2Fe(SO_4)_2{\cdot}6H_2O$ (20 µM); b) oxidation rates used to determine kinetic parameters of AspH for 2OG with hFX-Asn as the substrate; c) Michaelis–Menten plot for determination of the AspH k_{cat}^{app} and K_m^{app} values for 2OG with hFX-Asn as the substrate (values are in the Table below). The results reveal that the AspH k_{cat}^{app} value for 2OG with hFX-Asn as the substrate is similar to that reported for hFX-Asp,[16] whereas the AspH K_m^{app} value for 2OG with hFX-Asn as the substrate is ~4-fold greater than that reported for hFX-Asp.[16]

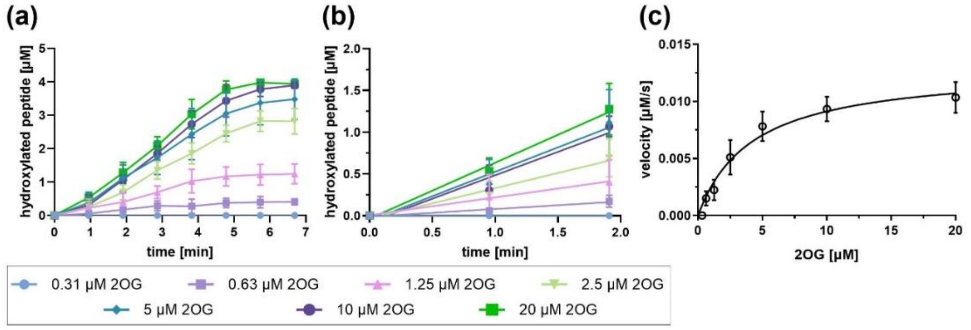

	substrate	k_{cat}^{app} $[s^{-1}]$	K_m^{app} [µM]	k_{cat}^{app}/K_m^{app} $[mM^{-1}s^{-1}]$
**2OG**	hFX-Asp	0.31 ± 0.03[16]	1.1 ± 0.4[16]	280 ± 110[16]
	hFX-Asn	0.26 ± 0.02	4.2 ± 1.0	62 ± 16

d) Time-course analysis of the AspH-catalysed oxidation of hFX-Asn (Figure 1) for the shown concentrations of $(NH_4)_2Fe(SO_4)_2{\cdot}6H_2O$ using 2OG (20 µM); e) hydroxylation rates used to determine kinetic parameters of AspH for Fe(II) with hFX-Asn as the substrate; f) Michaelis–Menten plot for determination of k_{cat}^{app} and K_m^{app} values for Fe(II) with hFX-Asn as the AspH substrate (values are in the Table below). The results reveal that the AspH k_{cat}^{app} and K_m^{app} values for Fe(II) with hFX-Asn as the substrate are similar, within experimental error, to those reported with hFX-Asp.[16]

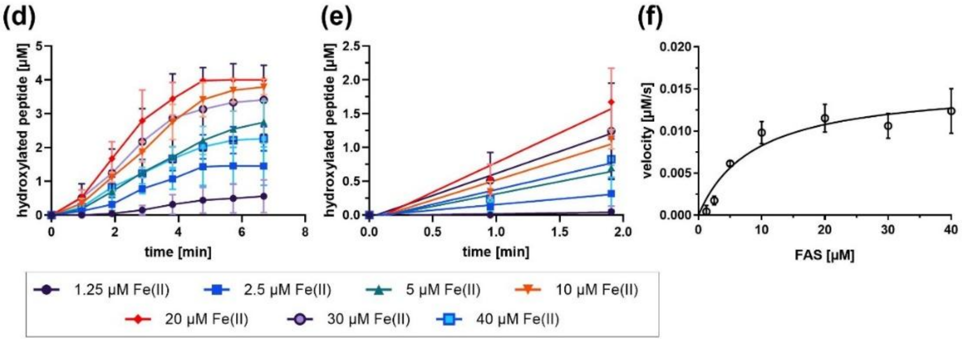

	substrate	k_{cat}^{app} $[s^{-1}]$	K_m^{app} $[\mu M]$	k_{cat}^{app}/K_m^{app} $[mM^{-1}s^{-1}]$
**Fe(II)**	hFX-Asp	0.27 ± 0.04[16]	4.3 ± 1.6[16]	63 ± 26[16]
	hFX-Asn	0.30 ± 0.03	8.2 ± 2.2	37 ± 11

g + h) Kinetic parameters of AspH for hFX-Asn were not determined because of substrate inhibition. Instead, direct competition assays were performed: hFX-Asn and an hFX-Asp-equivalent peptide were present in the same reaction vessel and oxidation was monitored using SPE-MS, as reported.[16] Because the masses of hFX-Asn and hFX-Asp were too similar to be robustly resolved, we employed an hFX-Asp derivative with 40 residues instead of 39 residues: a threonine residue was added to the C-terminus to match the reported hFX sequence,[17] i.e., the hFX-Asp(40) substrate. To exclude the possibility that the additional C-terminal residue of hFX-Asp(40) affected catalytic efficiency, we performed control experiments with the corresponding hFX-Asp and hFX-Asn(40) substrate pairs. The results reveal that AspH catalysed oxidation of: g) hFX-Asp(40) more efficiently than of hFX-Asn and h) hFX-Asp more efficiently than of hFX-Asn(40). The combined results indicate hFX-Asp is a more efficient AspH substrate than hFX-Asn, under the conditions employed.

Conditions: $AspH_{315-758}$ (0.05 μM), two substrate peptides (2.0 μM each), LAA (100 μM), 2OG (50 μM), and $(NH_4)_2Fe(SO_4)_2 \cdot 6H_2O$ (10 μM) in buffer (25 mM HEPES, pH 7.5, 50 mM NaCl, 20 °C). Data are means of independent triplicates (n = 3; mean ± SD).

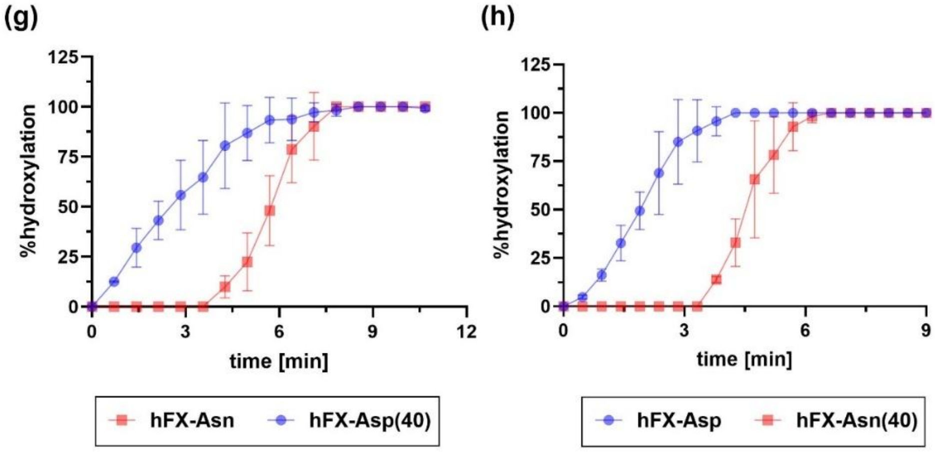

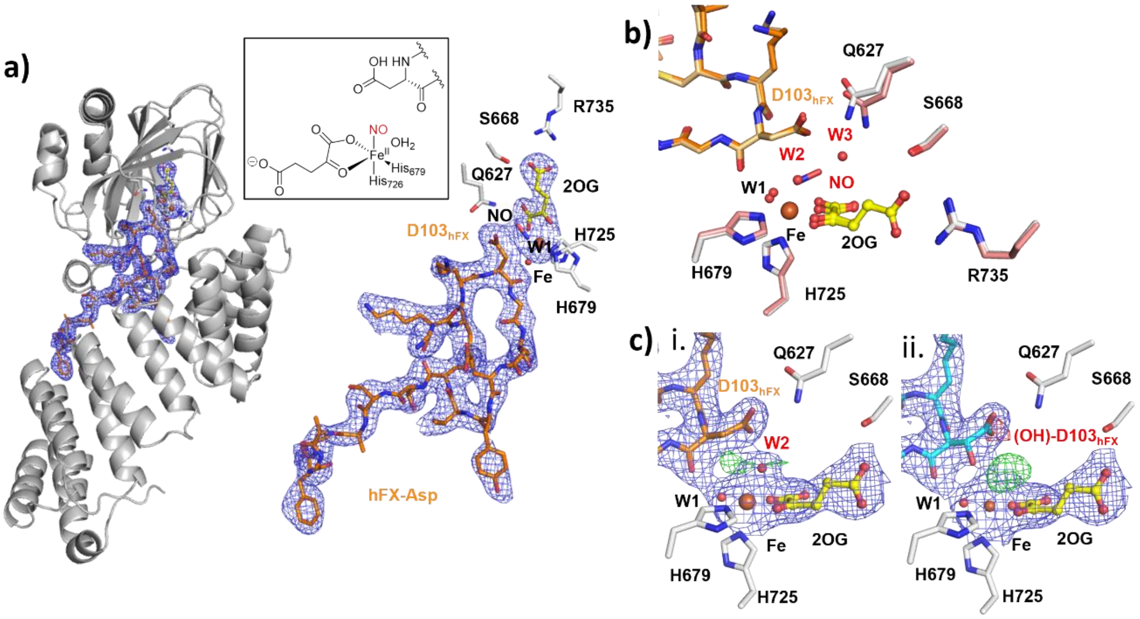

Supplementary Figure 5: Structure of the AspH:Fe:2OG:hFX-Asp complex after NO exposure. a) Ribbon structure and active site view with Polder omit maps (3.0 σ contour level, carved around substrate, Fe, 2OG, NO) of AspH:Fe:2OG:NO:hFX-Asp (PDB: 9HO3, 2.39 Å resolution). b) Active site superimposition of anaerobic AspH:Fe:2OG:hFX-Asp (PDB: 9FVZ, light orange and light pink) and NO-exposed AspH:Fe:2OG:hFX-Asp (PDB: 9HO3, bright orange and white) reveals binding of NO results in the replacement of W2 and W3, and shows movement of Q627 (0.7 Å of the carbonyl carbon); the side chain conformations of other residues are very similar to those in AspH:Fe:2OG:hFX-Asp (PDB: 9FVZ). c) Active site view of NO-exposed AspH:Fe:2OG:hFX-Asp (PDB: 9HO3), refined as (i) W2 instead of NO, and (ii) (3*R*)-OH-Asp103$_{hFX}$ instead of Asp103$_{hFX}$ and NO, showing 2mFo-DFc (1.0 σ contour level) in blue, overlaid with mFo-DFc (3.0 σ contour level) in green and red, revealing an improved fit upon modelling of NO in the AspH active site.

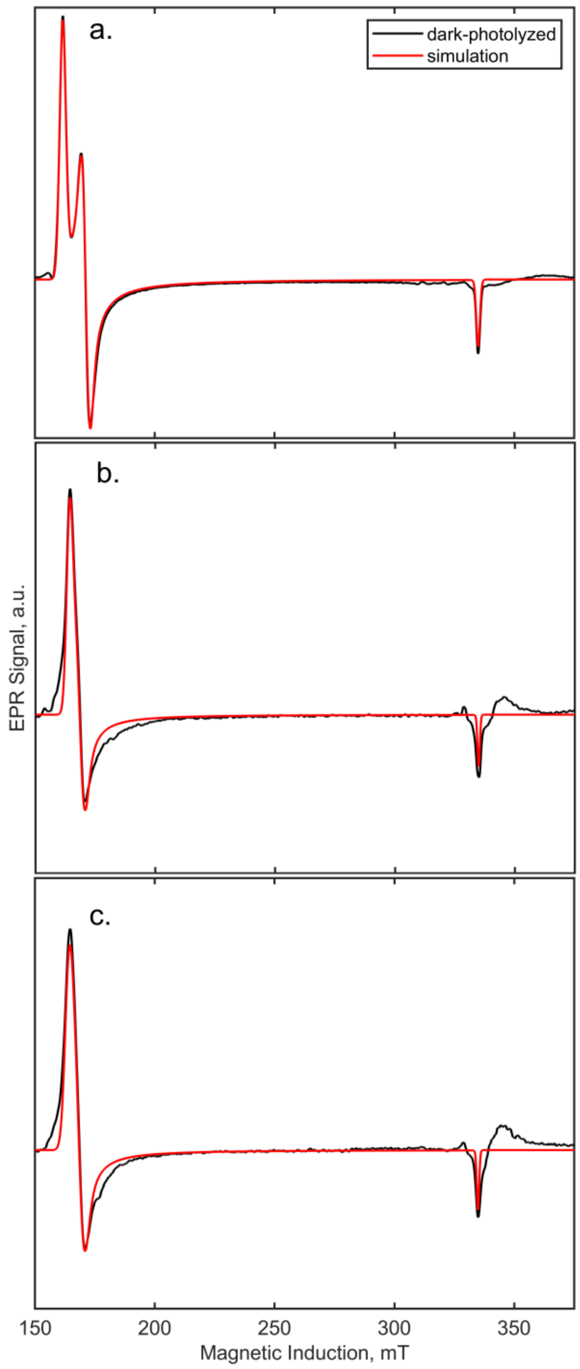

Supplementary Figure 6: EPR simulations of *hs* $[FeNO]^7$ signals in AspH with Fe(II), 2OG, NO and containing hFX-Asp/Asn peptide fragments. a) Simulation of S = 3/2 spin state of a 1 mM solution of AspH with 2OG, NO and hFX-Asp following subtraction of the dark state. b) Simulation of the S = 3/2 spin state of a microcrystalline slurry of AspH with 2OG, NO and hFX-Asp following subtraction of the dark state. c) Simulation of S = 3/2 spin state of a microcrystalline slurry of AspH with 2OG, NO and hFX-Asn following subtraction of the dark state. Simulation values are shown in Supplementary Table 1.

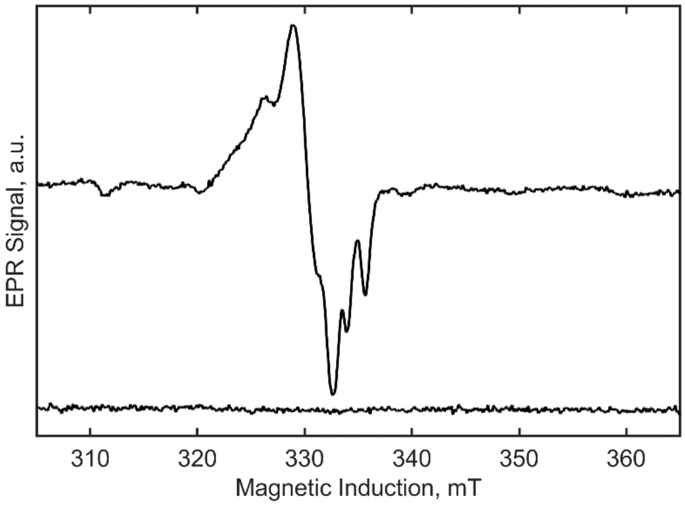

Supplementary Figure 7: X-band CW-EPR of AspH with Fe(II), 2OG, NO and hFX-Asp. The top trace represents the X-band CW-EPR at 50 K in the frozen solution state. The bottom trance represents the X-band CW-EPR in the frozen microcrystalline slurry, corresponding to data collected at 5 K as traces iii & ii, respectively, in main text Fig 3b. Conditions: microwave power 100 μW, 100 kHz field modulation 0.2 mT, and microwave frequency of 9.3836(1) GHz. Whilst Mn(II) ions were not introduced to our experiments, an apparent low-intensity Mn(II) signal is visible in our solution state complexes (upper trace) where a $Fe(NH_4)_2(SO_4)_2$ iron source was introduced. There was no evidence for an Mn(II) signal in our microcrystalline complex data (lower trace) were a $FeSO_4$ iron source was introduced, suggesting potential contamination in the $Fe(NH_4)_2(SO_4)_2$ source.

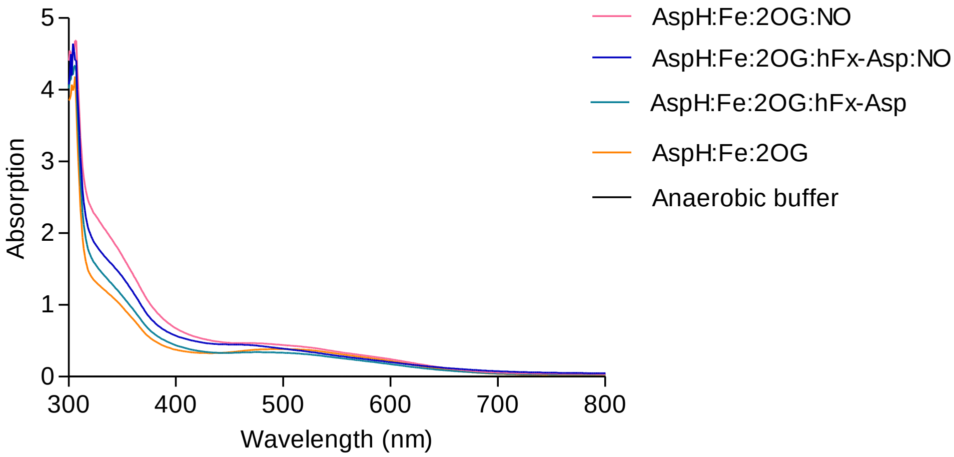

Supplementary Figure 8: UV-vis spectroscopic studies of NO binding to the AspH:Fe:2OG complex with hFX-Asp. AspH (1.0 mM) was incubated with ferrous ammonium sulphate (4.75 mM) and, where indicated, hFX-Asp (5 mM) in 50 mM HEPES pH 7.5, 150 mM NaCl, 20%$_{v/v}$ glycerol. Where indicated, samples were exposed to NO gas (1,000 ppm in N_2, 60 min). UV-Vis experiments were recorded using a final volume of 70 μL at 293 K, with a scan range from 200-800 nm (1 nm data interval) in a rectangular, quartz, ultra-micro, open top cuvette (2.5 x 5 mm, 10 mm pathlength. Samples were initially prepared in an anaerobic chamber (Belle Technology, UK); NO was introduced using a Schlenk line setup,[18] and sample collection in cuvettes equipped with a rubber septum seal.

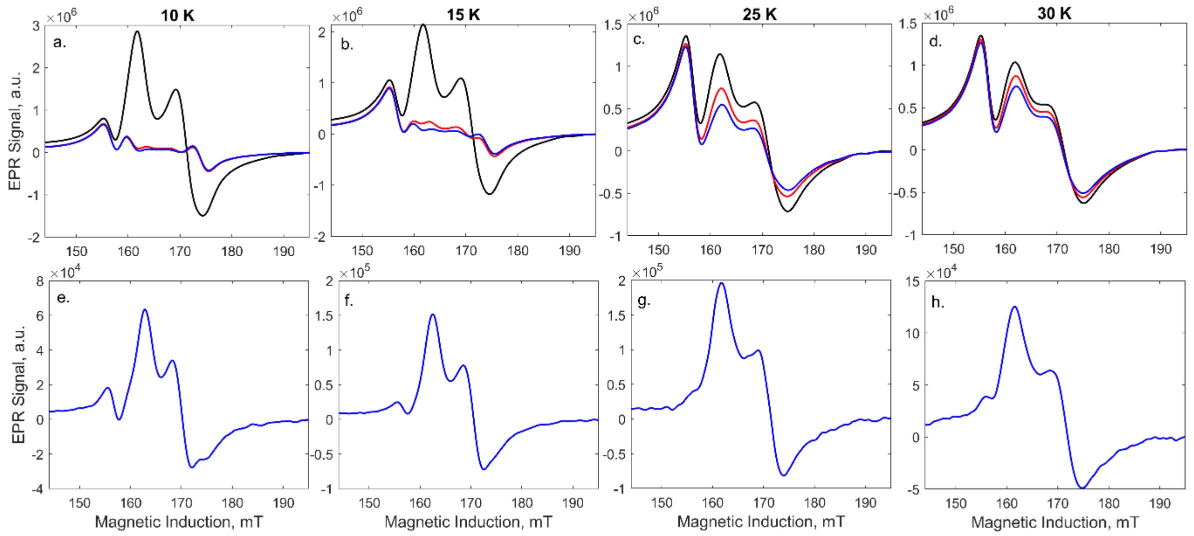

Supplementary Figure 9: EPR photolysis experiments of AspH with Fe(II), 2OG, NO and the hFX-Asp peptide fragment. Photolysis differences and recovery analyses with AspH, Fe(II) 2OG, NO and hFX-Asp, analysed using X-band CW-EPR at four temperatures: a+e) 10 K; b+f) 15 K; c+g) 25 K; and d+h) 30K. The pre-photolysis spectra are in black, the extent of photolysis achieved to quasi-steady state is in blue, and recovery end point is in red for panels a-d. The difference of the extent of recovery and the photolyzed condition are shown in e-h. Conditions: microwave power 5 mW, 100 kHz field modulation 0.2 mT, and microwave frequency of 9.3837(2) GHz.

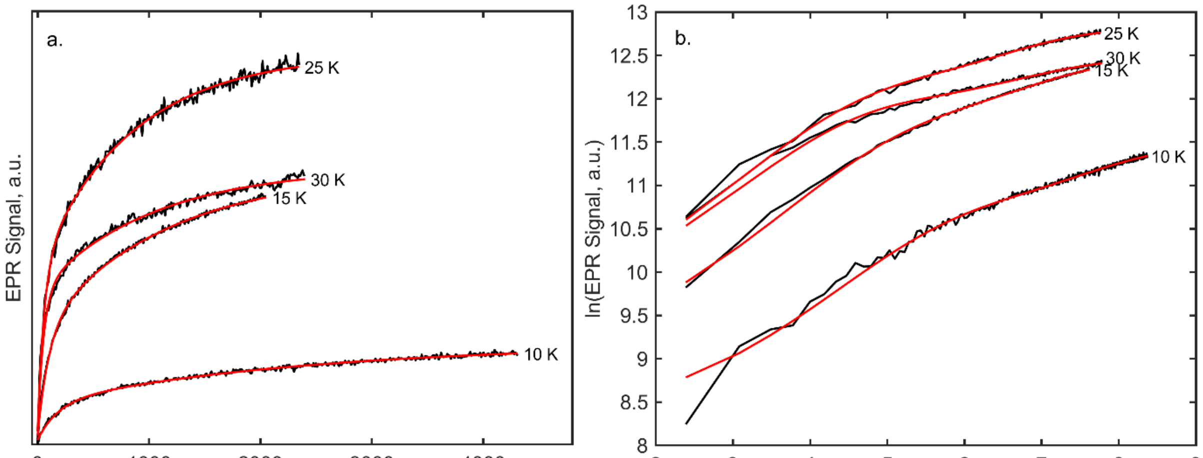

Supplementary Figure 10: Fits of biexponential functions (red) to post-photolysis EPR signal recovery for (a) linear and (b) log-log scales. Data are taken as the maximum minus minimum of Fig S9. Zero is defined as the initial point on lamp switch-off after quasi steady-state photolysis. Conditions are given in the Supplementary Figure 9 legend.

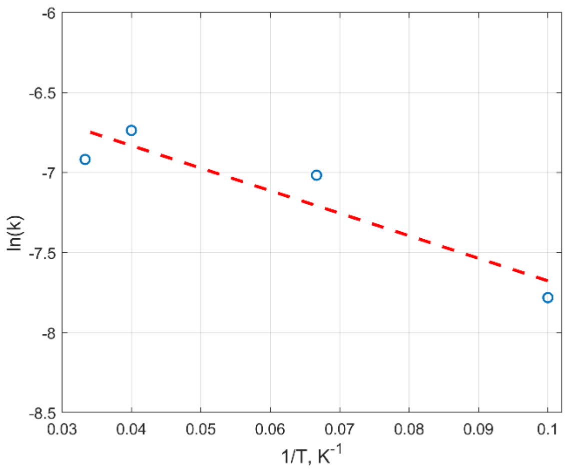

Supplementary Figure 11: Linear fit (red dashed) to the shortest of two components in biexponential fits of Supplementary Figure 10 (blue dots). The fit gives an Ea of 120 J/mol, whilst fit to the longer biexponential component gives an Ea of 150 J/mol.

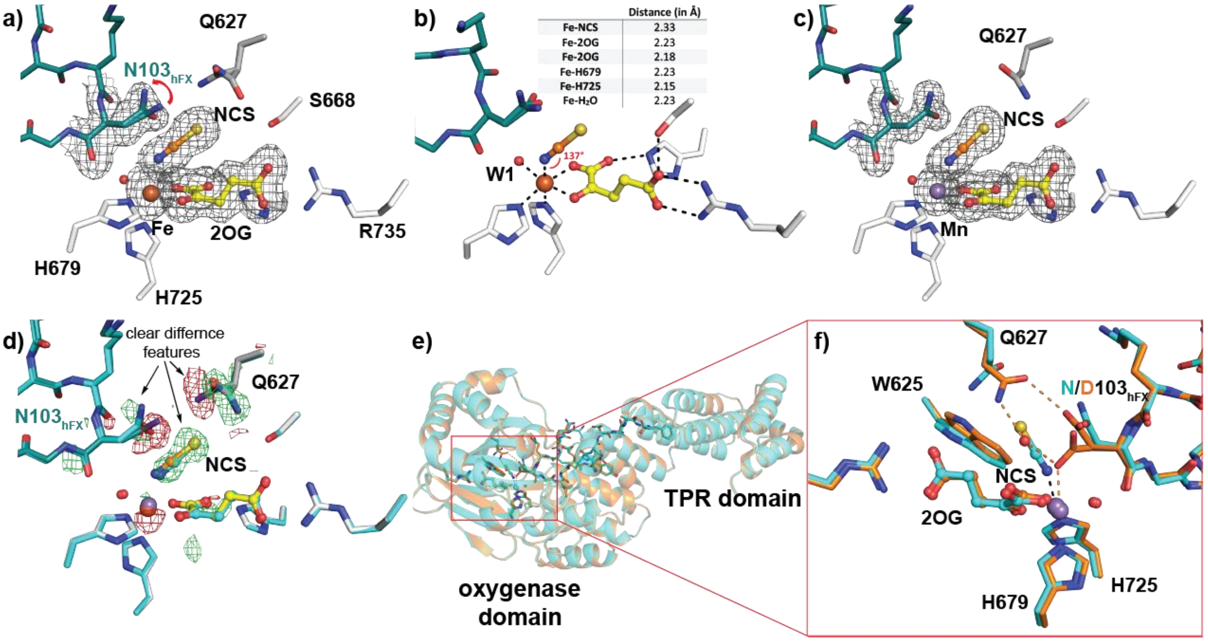

Supplementary Figure 12: Views from structures of the AspH:Fe/Mn:2OG:hFX-Asn complexes in the presence of thiocyanate ion (NCS^-). a) Polder omit map (3.0 σ contour level, carved around hFX-Asn, Fe, 2OG, NCS) of AspH:Fe:2OG:hFX-Asn in the presence of thiocyanate (PDB: 9FVU, 1.70 Å resolution, anaerobic) reveals partial binding via the NCS-nitrogen as an isothiocyanate anion (~70% occupancy) *trans* to H725. Note that the steric impact of NCS binding causes rearrangement of the substrate residue Asn103 (present in two conformations: conf. A re-arranged conformation (+NCS^-, ~70% occupancy) and conf. B original conformation (-NCS^-, ~30% occupancy), as illustrated by a red arrow. b) Key interactions, distances (Å) and angles, in the active site of AspH:Fe:2OG:hFX-Asn:NCS (PDB: 9FVU) complex. c) Polder omit map (3.0 σ contour level, carved around hFX-Asn, Mn, 2OG, NCS) of AspH:Mn:2OG:hFX-Asn in presence of thiocyanate (PDB: 9FVV, 1.60 Å resolution) implies complete NCS^- binding (Mn–N–C angle: 137°). Isothiocyanate binding causes Asn103 and Q627 to adopt an alternate conformation. Mn is shown as an orange sphere. Note: The higher isothiocyanate occupancy in the Mn complex may reflect the reduced coordination flexibility and larger ionic radius of Mn(II) compared to Fe(II), the former of which apparently favours stronger and less flexible ligand binding. By contrast, the catalytically relevant Fe(II) centre in AspH displays a more flexible coordination environment, consistent with the partial occupancy of water and other ligands observed in the Fe-bound

structures. d) F_{obs} − F_{obs} isomorphous difference map of the active site superimposition of AspH:Fe:2OG:hFX-Asn:NCS (PDB: 9FVU, shown as grey sticks with the substrate in teal) relative to AspH:Mn:2OG:hFX-Asn:NCS (PDB: 9FVV, shown in cyan) displayed at +3.0 σ (green) and −3.0 σ (red) contour levels supporting partial binding of NCS^- in the Fe structure with resulting rearrangement of Asn103 and Gln 627. e + f) Superimposition of AspH:Mn:2OG:hFX-Asp (PDB: 8RE9,[15] shown in orange) and AspH:Mn:2OG:hFX-Asn:NCS (PDB: 9FVV, shown in cyan) structures revealing distinct differences in the W625, Q627 and $X103_{hFX}$ orientations following isothiocyanate binding.

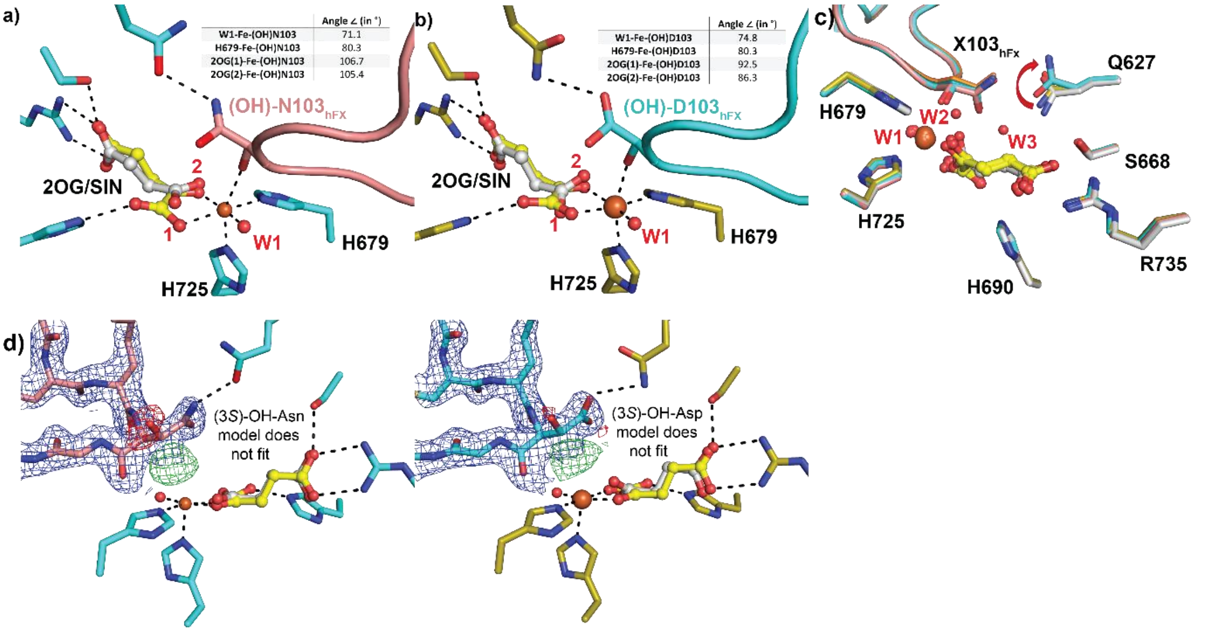

Supplementary Figure 13: Structures of the AspH:Fe:2OG complexes with the hFX-Asp/Asn substrate after O_2 exposure. a and b) Key interactions and angles of the Fe, 2OG and product coordinating residues in the: a) AspH:Fe:2OG:hFX-(OH)Asn (PDB: 9FVW) and b) AspH:Fe:2OG:hFX-(OH)-Asp (PDB: 9FVY) structures. The hydroxy group of the hydroxylated Asn103 and Asp103 residues occupies the Fe coordination site opposite H725, forming a distorted octahedral Fe-coordination geometry preventing coordination of H_2O to Fe *trans* to H725. c) Active site superimposition of anaerobic AspH:Fe:2OG:hFX-Asn (PDB: 9FVX), AspH:Fe:2OG:hFX-Asp (PDB: 9FVZ), O_2 exposed AspH:Fe:2OG:hFX-(OH)Asn (PDB: 9FVW, main chain RMSD = 0.281 Å compared to hFX-Asn) and O_2-exposed AspH:Fe:2OG:hFX-(OH)Asp (PDB: 9FVY, main chain RMSD = 0.266 Å compared to hFX-Asp) complexes revealing minor differences in the active site Fe, 2OG, and substrate/product orientations. Note there is a difference in the Q627 side chain conformation in the substrate/product complexes. d+e) $2mF_0$-DF_c (blue) and mF_o-DF_c (for positive density: green; for negative density: red) of modelling attempts using (3*S*)-OH-Asn and (3*S*)-OH-Asp in AspH:Fe:2OG:hFX-(OH)Asn (PDB: 9FVW) and AspH:Fe:2OG:hFX-Asp (PDB: 9FVY), respectively, revealing clear difference density supporting the introduction of the hydroxy group with the (3*R*)-stereochemistry, as anticipated based on pioneering work.[19,20]

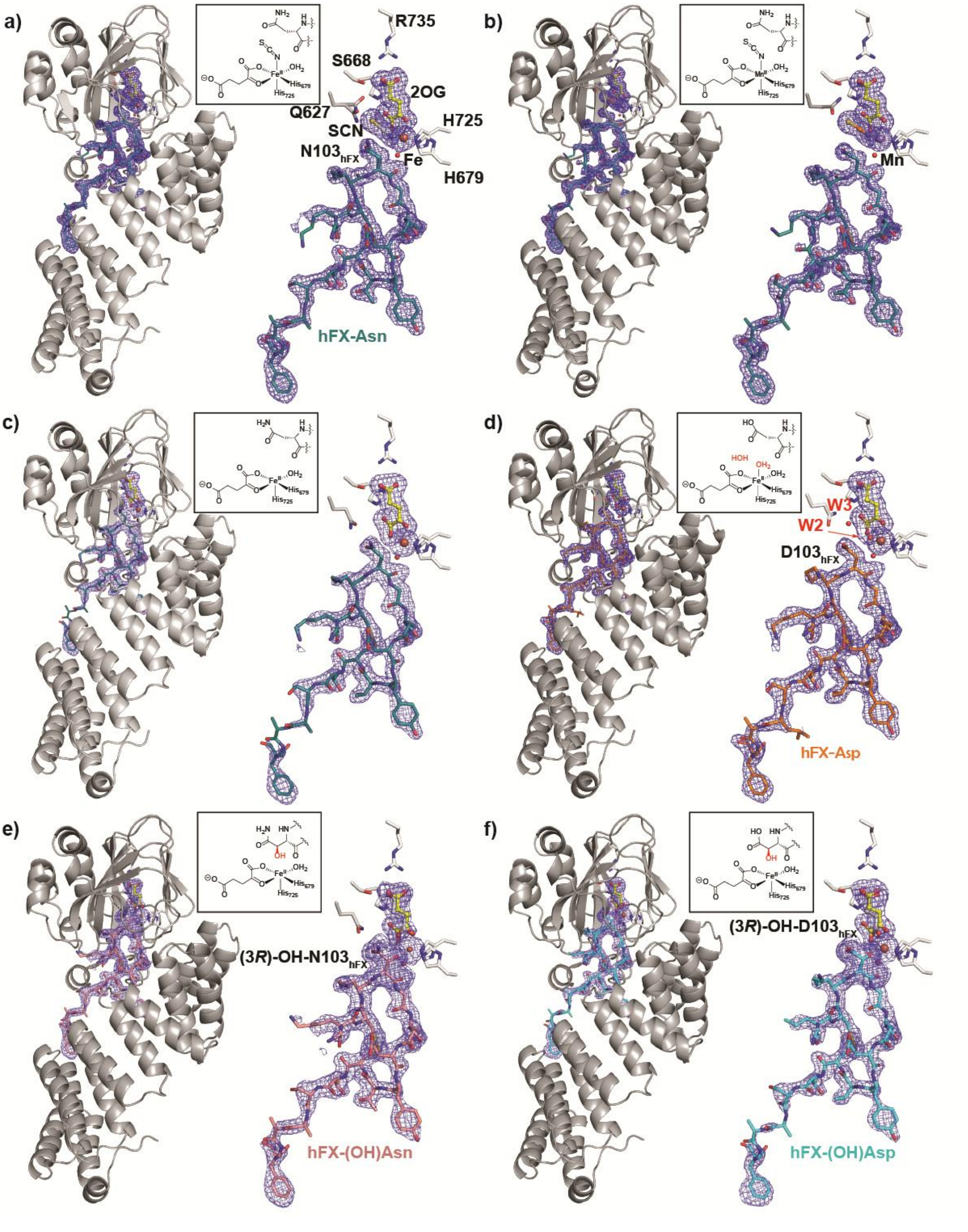

Supplementary Figure 14: Polder omit maps of the AspH:Fe:2OG/succinate complexes with the hFX-Asp/Asn substrate under anaerobic and O_2-exposed conditions in the presence and absence of thiocyanate ion (NCS^-). Ribbon structure and active site view on Polder omit maps (3.0 σ contour level, carved around the hFX-Asp/Asn, Fe, 2OG, NCS) of: a) AspH:Fe:2OG:hFX-Asn:NCS (PDB: 9FVU, 1.70 Å resolution, teal), b) AspH:Mn:2OG:hFX-Asn:NCS (PDB: 9FVV, 1.60 Å resolution), c) AspH:Fe:2OG:hFX-Asn (PDB: 9FVX, 1.90 Å resolution), d) AspH:Fe:2OG:hFX-Asp (PDB: 9FVZ, 1.95 Å resolution, orange), e) AspH:Fe:2OG/SIN:hFX-(OH)Asn (PDB: 9FVW, 1.90 Å resolution, purple) and f) AspH:Fe:2OG/SIN:hFX-(OH)Asp (PDB: 9FVY, 1.90 Å resolution, cyan). The insets show the active site metal coordination and residue-103.

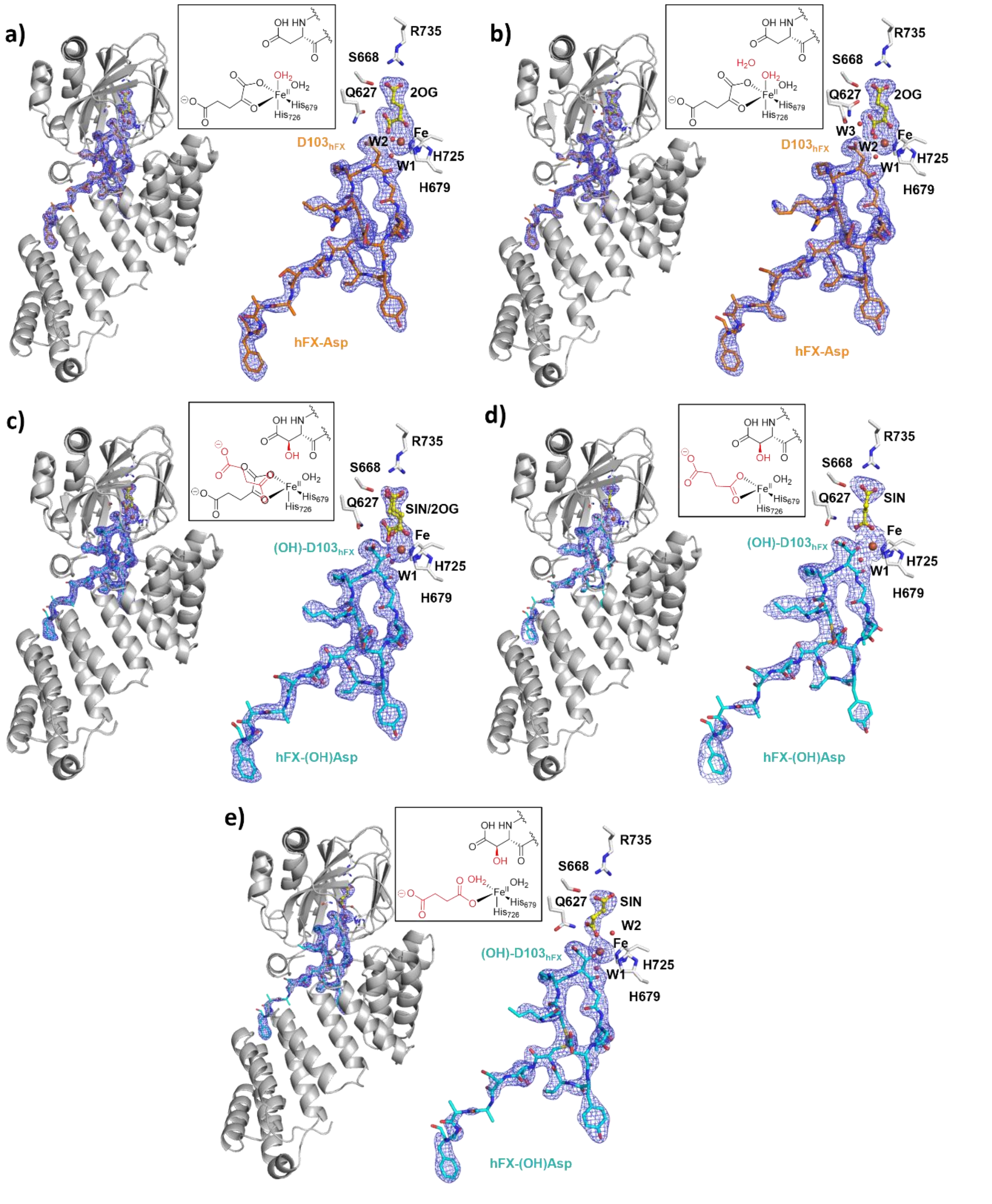

Supplementary Figure 15: Polder omit maps of the room temperature structures of AspH:Fe:2OG/succinate complexes with the hFX-Asp substrate under anaerobic and O_2-exposed conditions. Ribbon structure and active site view with Polder omit maps (3.0 σ contour level, carved around the hFX-Asp, Fe, 2OG/2OG) of: a) anaerobic AspH:Fe:2OG:hFX-Asp (excess Fe, PDB: 9FW0, 1.95 Å resolution, orange), b) anaerobic AspH:Fe:2OG:hFX-Asp (no excess Fe, PDB: 9HO2, 1.83 Å resolution, orange), c) AspH:Fe:2OG/SIN:hFX-(OH)Asp following 1.5 s O_2 exposure (PDB: 9HO1, 1.85 Å resolution, cyan), d) AspH:Fe:SIN:hFX-(OH)Asp following 2-6 h exposure to air (PDB: 9NHZ, 2.40 Å resolution, cyan), and e) AspH:Fe:SIN:hFX-(OH)Asp following 12-24 h exposure to air (PDB: 9HO0, 2.14 Å resolution, cyan) structures.

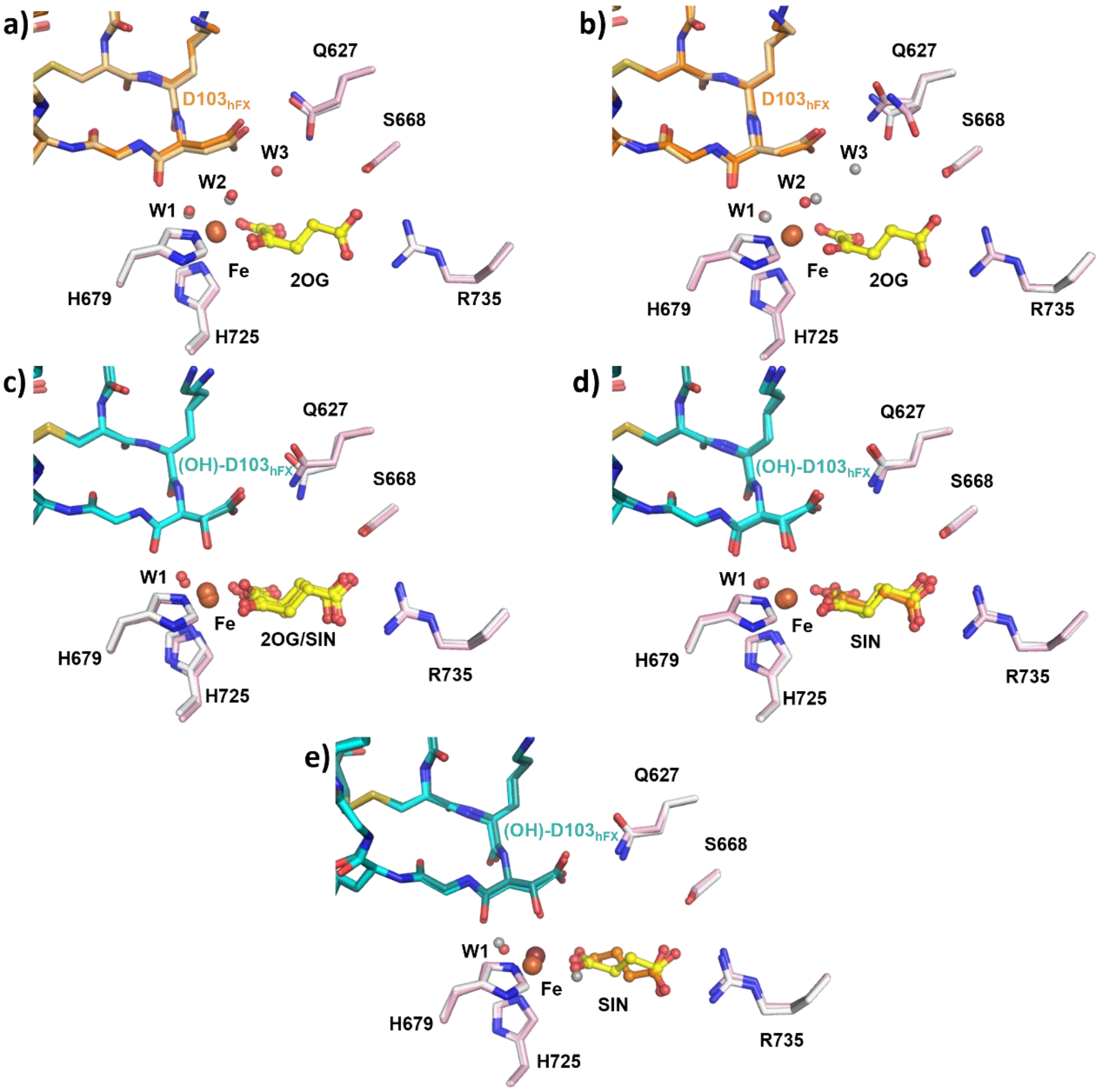

Supplementary Figure 16: Superimpositions of AspH:Fe:2OG/succinate complex structures with the hFX-Asp substrate under anaerobic and O_2-exposed conditions. a) Superimposition of the active site of the anaerobic AspH:Fe:2OG:hFX-Asp complex obtained under cryogenic-conditions (PDB: 9FVZ; orange and grey) with a room temperature structure (PDB: 9FVZ; light orange and light pink) showing overall high similarity (RMSD = 0.240 Å). Note, W3 was only observed under cryogenic conditions. b) Superimposition of the active site of the room temperature anaerobic AspH:Fe:2OG:hFX-Asp complex obtained with excess iron (PDB: 9FW0; orange and pink) with a structure obtained without excess iron (PDB: 9HO2; light orange and light pink) showing overall high similarity (RMSD = 0.140 Å). Note, W3 was only observed in the structure without excess iron, and a tilt in the Q627 sidechain and a second conformation of Q627 is observed (0.9 Å from the carbonyl carbon in conformation 1, and 1.4 Å from the carbonyl carbon in conformation 2). c) Superimposition of the active site of the O_2-exposed AspH:Fe:2OG:hFX-Asp complex obtained under cryogenic-conditions (PDB: 9FVY; cyan and grey) with a room temperature structure obtained after 1.5 s O_2 exposure (PDB: 9HO1; deep teal and light pink) showing overall high similarity (RMSD = 0.296 Å). d) Superimposition of the active site of the O_2-exposed AspH:Fe:2OG:hFX-Asp complex obtained at room temperature after 1.5 s O_2 exposure (PDB: 9HO1; cyan and grey) with a structure obtained at room temperature after 2-6 h exposure to air (PDB: 9NHZ; deep teal and light pink) showing overall high similarity (RMSD = 0.162 Å), but with the latter manifesting only succinate (orange) with no evidence for 2OG (yellow). e) Superimposition of the active site of the O_2-exposed AspH:Fe:2OG:hFX-Asp complex obtained at room temperature after 2-6 h exposure to air (PDB: 9NHZ; cyan and grey) with a structure obtained at room temperature after 12-24

h exposure to air (PDB: 9HO0; deep teal and light pink) showing overall high similarity (RMSD = 0.171 Å). Note, the conformation and position of succinate (orange) is different in the two structures and an additional water (gray) is bound to the active site Fe of the 12-24 h exposure to air structure (PDB: 9HO0).

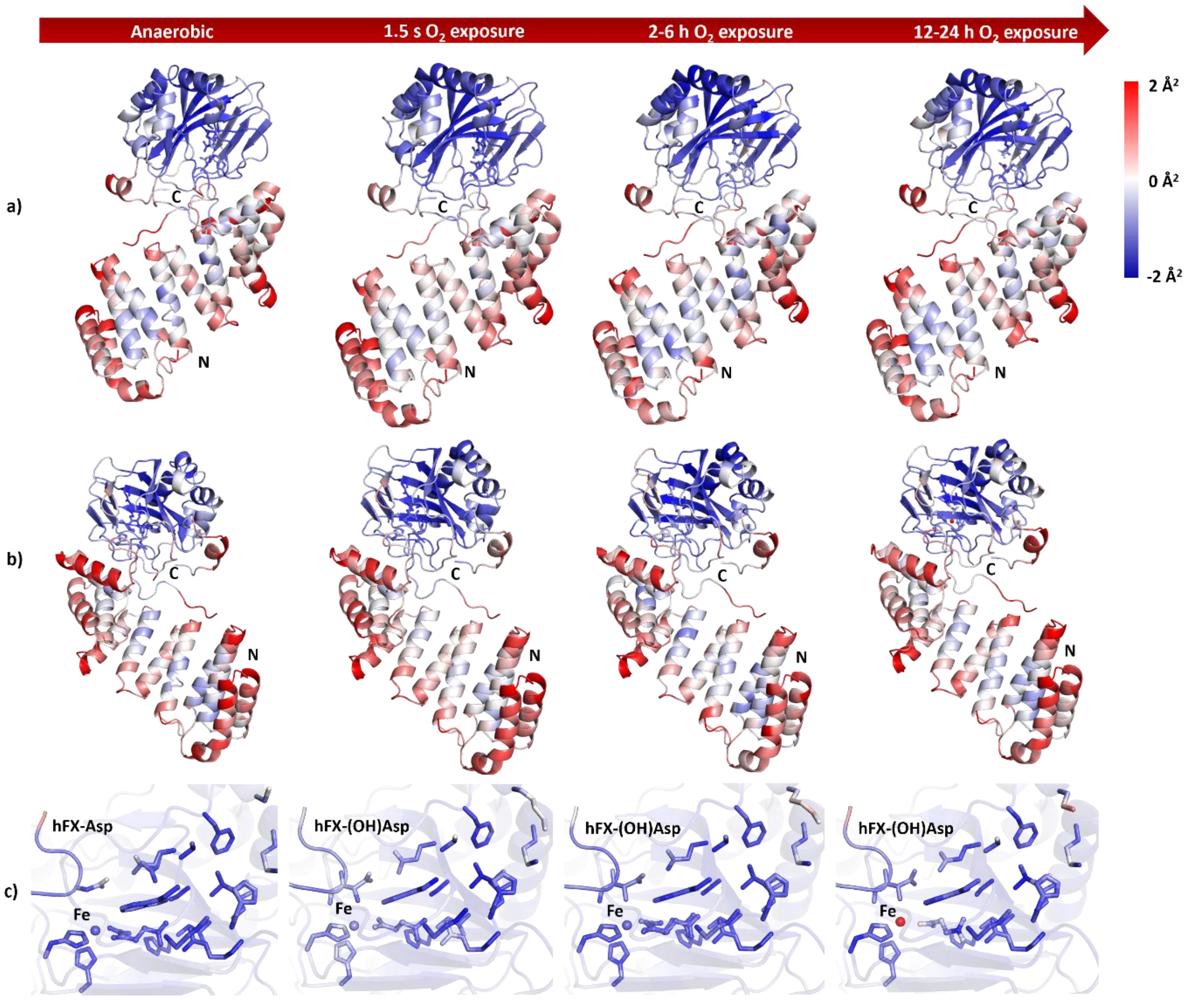

Supplementary Figure 17: Analysis of normalised B-factors in AspH:Fe:2OG/succinate complexes with the hFX-Asp peptide obtained under anaerobic and O_2 exposed conditions. a) View of AspH:Fe:2OG/succinate:hFX-Asp complexes with B-factors for backbone atoms normalised (using PyMOL). b) Reverse view of AspH:Fe:2OG/succinate:hFX-Asp complexes with B-factors for backbone atoms normalised. c) View of the active site and the proposed 2OG binding tunnel tunnel of AspH:Fe:2OG/succinate:hFX-Asp complexes with the B-factors for side chain atoms normalised. B-factors for backbone atoms are shown as cartoons and coloured according to B-factors from blue (low B-factor) to red (high B-factor). B-factors for side chain atoms are shown as sticks and coloured according to B-factors from blue (low B-factor) to red (high B-factor). The different structures are very similar,, both in the backbone atoms and the side chain atoms in the catalytic domain. Details of B-factor analyses are given in Materials and Methods.

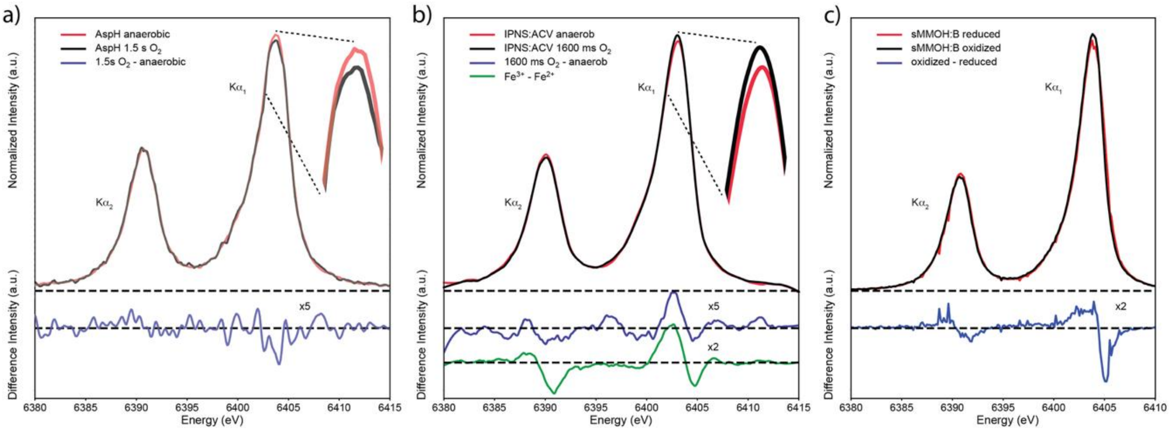

Supplementary Figure 18: Fe Kα XES analysis of AspH compared with IPNS and sMMO. a) The spectra obtained for anaerobic AspH and after 1.5 s exposure to O_2 are shown after area normalization, together with the resulting difference spectrum for the O_2-exposed anaerobic state (blue). The $K\alpha_1$ peak region is enlarged in the inset. The difference spectrum shows some reduction in the energy of the $K\alpha_1$ peak maximum, correlating with the observation that the anaerobic sample shows a higher $K\alpha_1$ peak intensity compared to the O_2-exposed form. b) The area normalized spectra obtained previously for the IPNS:ACV complex under anaerobic conditions (red), as well as after 1.6 s O_2 incubation (black), are shown, together with the difference spectrum (blue) and the difference for the Fe^{2+} and Fe^{3+} standards.[18] The difference spectrum correlates with the shift observed in the Fe^{3+} - Fe^{2+} standards, indicating that after 1.6 s O_2 incubation the mononuclear Fe site in IPNS is predominantly oxidized to the Fe^{3+} form. The inset shows the $K\alpha_1$ peak region for both IPNS spectra, showing that the O_2-exposed form has a higher peak maximum compared to the anaerobic form. This is reversed to the observation for the AspH sample. c) The area normalized spectra previously obtained for the di-nuclear Fe site in soluble methane monooxygenase (sMMO) in the di-ferrous (reduced, red) and di-ferric (oxidized, black) state.[21] The difference spectrum (blue) indicates a trend roughly similar to what was observed for IPNS and for the Fe^{3+} - Fe^{2+} reference sample. eV = electron volt, a. u. = arbitrary units.

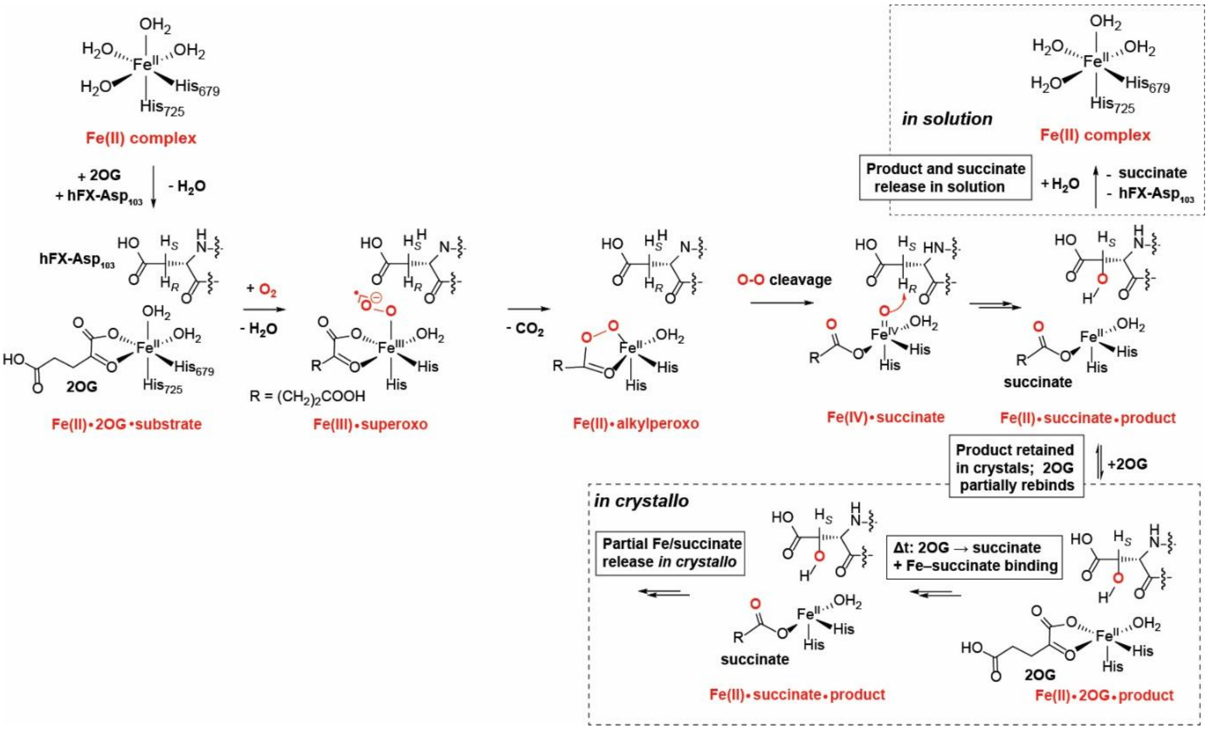

Supplementary Figure 19: Schematic view of the progression of AspH catalysis in solution and *in crystallo*. Proposed catalytic cycle of AspH highlighting differences between turnover in solution and within crystals. The reaction starts with binding of Fe(II), 2OG, and substrate, followed by O_2 activation, to form the Fe(III) superoxo, then Fe(II) alkylperoxo intermediates, O-O bond cleavage, and generation of an Fe(IV) succinate species that hydroxylates the substrate. In solution, subsequent release of succinate and product yields the resting Fe(II) complex. In crystallo, product release is partially restricted, resulting in retention of the large hydroxylated hFX-Asp substrate (due to crystal lattice restraints), while the smaller cosubstrate 2OG can partially rebind to the Fe centre, leading to the mixed Fe succinate and Fe 2OG bound state observed in the crystal structures. Over time residual 2OG is converted to succinate due to progressive solution-phase turnover and only succinate is available for binding to the Fe centre as observed in the >2h O_2 exposure data sets.

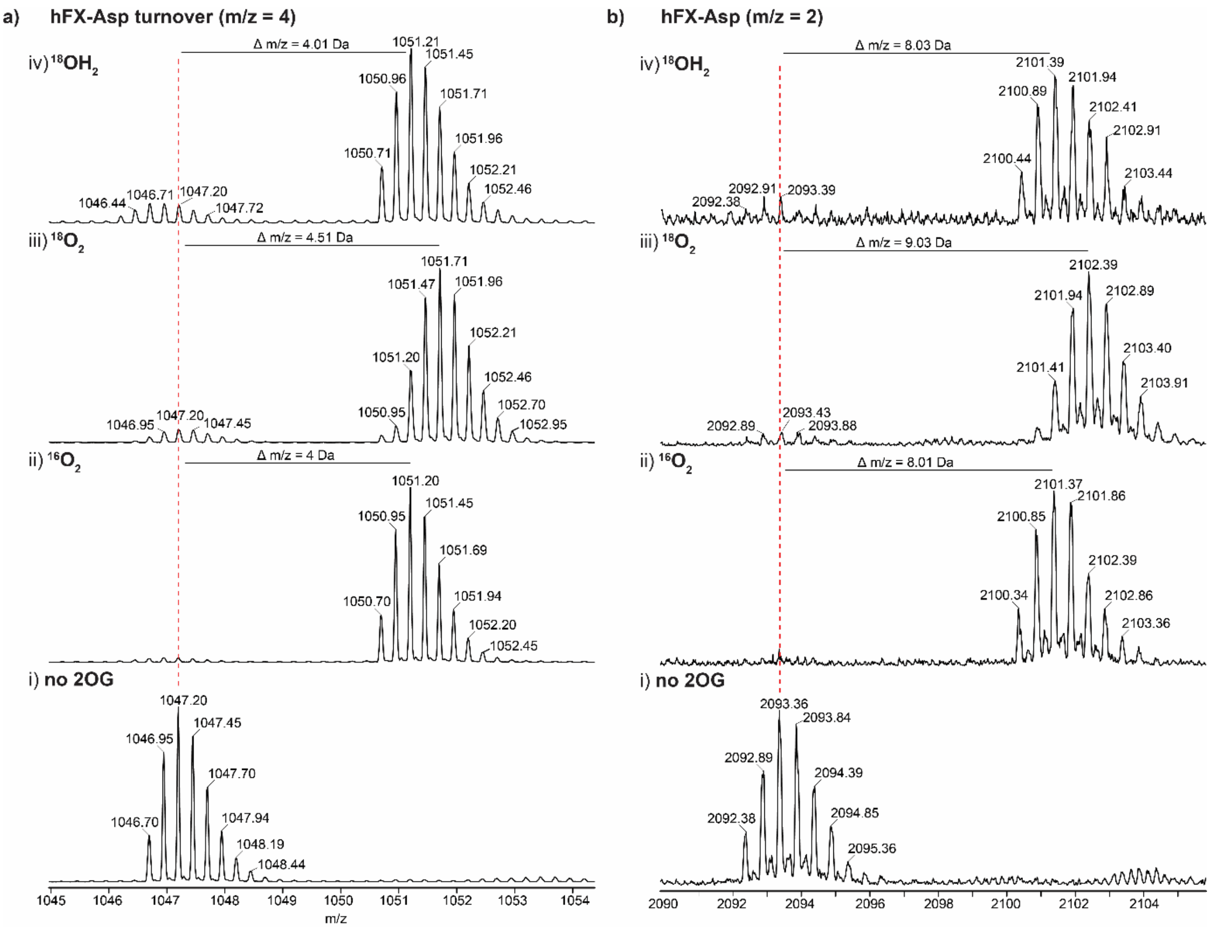

Supplementary Figure 20: $H_2{}^{18}O$ and $^{18}O_2$ labelling experiments with AspH and hFX-Asp analysed using LC–MS. Mass spectra after 180 min incubation of AspH (1 µM) with hFX-Asp (20 µM), sodium L-ascorbate (100 µM), 2OG (200 µM), and $(NH_4)_2Fe(SO_4)_2$ (20 µM) in 50 mM HEPES (pH 7.5) + 50 mM NaCl. The spectra show the regions containing: a) $[M+4H]^{4+}$ and b) $[M+2H]^{2+}$ substrate and product mass ions. Calculated substrate masses: 1047.2 Da ($[M+4H]^{4+}$) and 2093.4 Da ($[M+2H]^{2+}$). Calculated ^{16}O product mass: 1051.2 Da ($M+4H]^{4+}$) and 2101.4 Da ($[M+2H]^{2+}$). Calculated ^{18}O product masses: 1051.7 Da ($M+4H]^{4+}$) and 2102.4 Da ($[M+2H]^{2+}$).

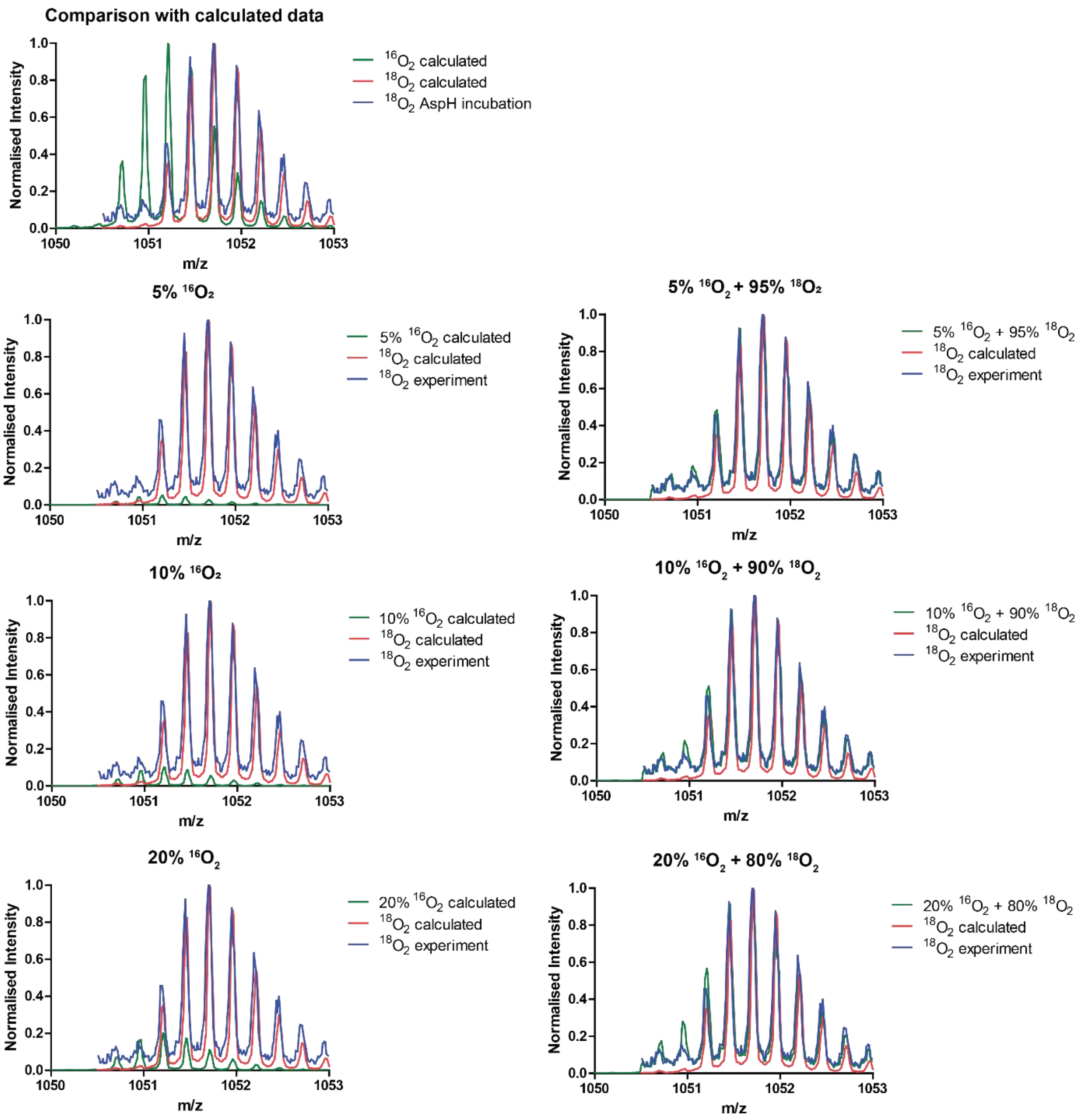

Supplementary Figure 21: Analysis of $^{18}O_2$ isotope incorporation and assessment of $^{16}O_2$ contamination. Quantitative analysis of $^{18}O_2$ isotope-labelling experiments was performed to assess the extent of isotope incorporation into the hFX-Asp substrate. Isotopic envelopes were calculated for reactions carried out under $^{16}O_2$ (m/z increase of 4.0 Da at charge state 4^+) and $^{18}O_2$ (m/z increase of 4.5 Da at charge state 4^+) conditions. Simulated distributions representing admixtures of 5%, 10%, and 20% $^{16}O_2$ were generated to model potential contamination from atmospheric oxygen. Comparison of these calculated distributions (green and red traces) with the experimental spectra (blue traces) recorded at pH 7.5 shows that the data are best described by a model containing approximately 5–10% $^{16}O_2$. Considering the stated 95% isotopic purity of the commercial $^{18}O_2$ gas, this level of $^{16}O_2$ signal is consistent with minor contamination from the gas source or ambient air. Control reactions performed with $^{18}OH_2$ showed no evidence for ^{18}O isotope incorporation.

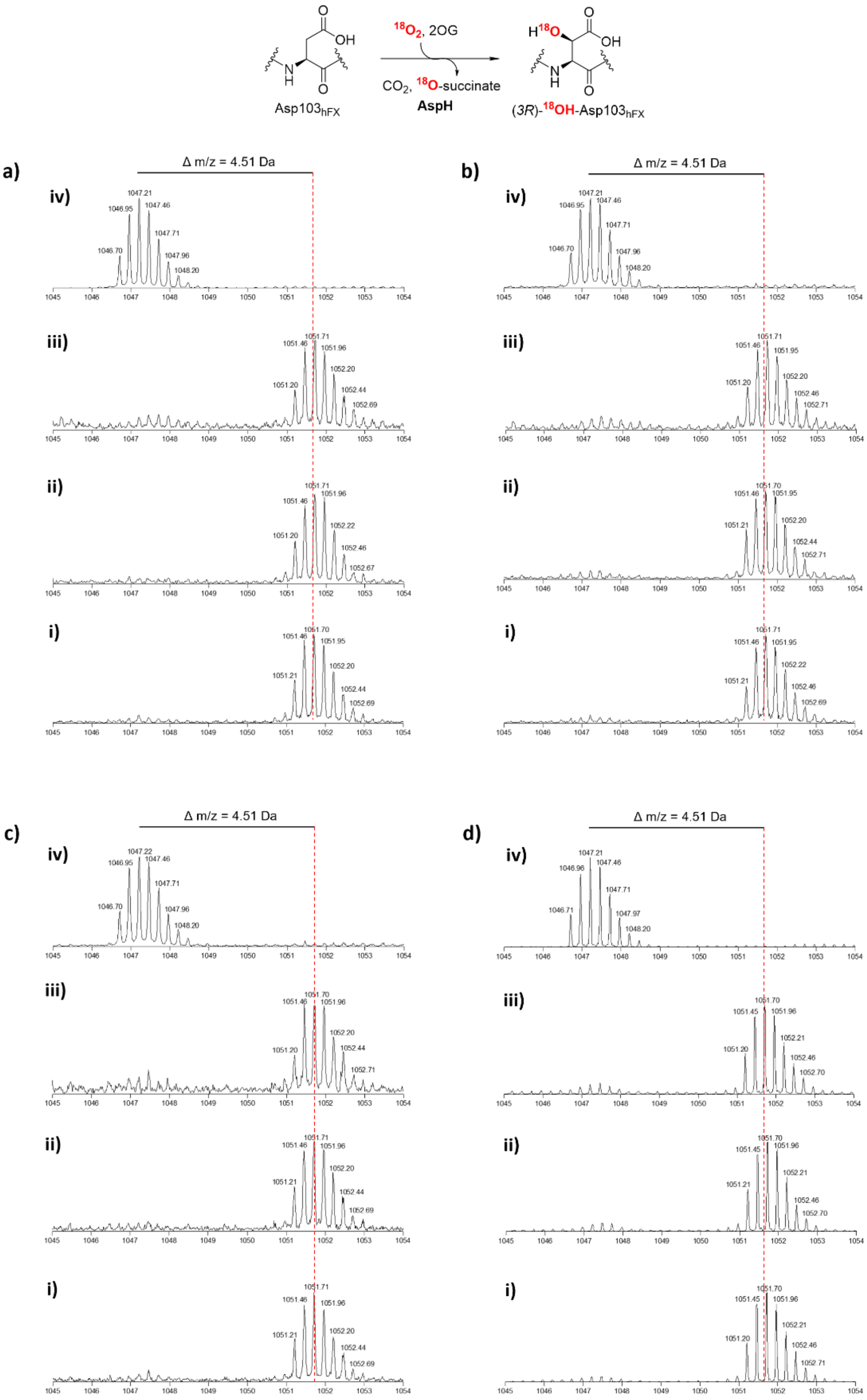

Supplementary Figure 22: $^{18}O_2$ labelling experiments with AspH and hFX-Asp using different 2OG concentrations and pH. Mass spectra following overnight incubation of AspH (1 µM) with hFX-Asp (20 µM), sodium L-ascorbate (100 µM), 2OG [i) 20 µM, 1 equiv.; ii) 50 µM, 2.5 equiv.; iii) 100 µM, 5 equiv.; iv) no 2OG], and $(NH_4)_2Fe(SO_4)_2$ (20 µM) in 50 mM HEPES (pH 7.0 – pH 8.5) + 50 mM NaCl. The spectral regions show the $[M+4H]^{4+}$ substrate and product masses at varying pH values: a) pH 7.0, b) pH 7.5, c) pH 8.0 and d) pH 8.5. Calculated substrate mass: 1047.2 Da ($[M+4H]^{4+}$). Calculated ^{18}O product mass: 1051.7 Da ($M+4H]^{4+}$).

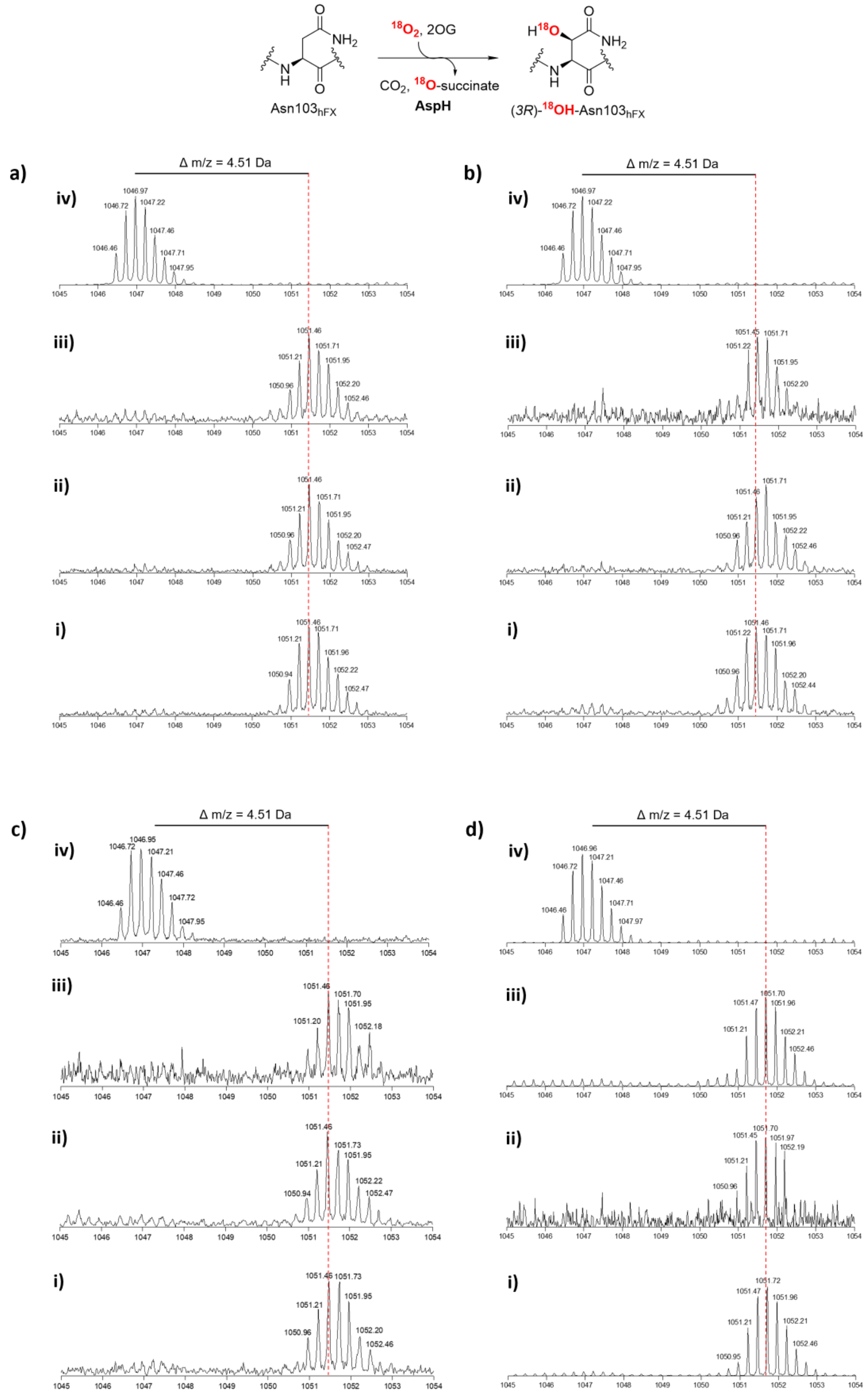

Supplementary Figure 23: $^{18}O_2$ labelling experiments with AspH and hFX-Asn using different 2OG concentrations and pH. Mass spectra following overnight incubation of AspH (1 µM) with hFX-Asn (20 µM), sodium L-ascorbate (100 µM), 2OG [i) 20 µM, 1 equiv.; ii) 50 µM, 2.5 equiv.; iii) 100 µM, 5 equiv.; iv) no 2OG], and $(NH_4)_2Fe(SO_4)_2$ (20 µM) in 50 mM HEPES (pH 7.0 – pH 8.5) + 50 mM NaCl. The spectral regions show the $[M+4H]^{4+}$ substrate and product masses at varying pH values: a) pH 7.0, b) pH 7.5, c) pH 8.0 and d) pH 8.5.

Supplementary Table 1: EPR simulation parameters of *hs* $[FeNO]^7$ signals in AspH with 2OG, NO and hFX-Asp/Asn substrate. [a]Broad distributions of parameter values visible at low intensity are also present. [b]Effective g-values are used as fitting data, axial g-values significantly improve fit over an isotropic g-value. [c]Zero-Field Splitting rhombicity λ=E/D, where the arbitrarily-large D is set to 100 cm^{-1}.

Sample	$S' = 1/2$ g_1, g_2, g_3	l.w.(1,2,3) / MHz	$S = 3/2$ $g_\perp, g_\parallel$ [b]	$S = 3/2$, λ[b,c]
hFX-Asp, solution	4.15, 3.915, 2.0017	165, 182, 41	2.016, 2.0022	0.019
hFX-Asp, microcrystals [a]	4.082, 3.972, 2.0016	165, 206, 17	2.014, 2.0021	0.0091
hFX-Asn, microcrystals [a]	4.082, 3.974, 2.0016	221, 238, 17	2.014, 2.0021	0.0089

Supplementary Table 2: Data collection and Refinement statistics.

**Datasets**	**AspH:Fe:2OG:hFX-Asn** (PDB ID: 9FVX)	**AspH:Fe:2OG:hFX-Asp** (PDB ID: 9FVZ)	**AspH:Fe:2OG/succinate:hFX-(OH)Asn** (PDB ID: 9FVW)	**AspH:Fe:2OG/succinate:hFX-(OH)Asp** (PDB ID: 9FVY)	**AspH:Fe:2OG:hFX-Asn:SCN** (PDB ID: 9FVU)
**Data Collection (T in K)**	MX (100)	MX (100)	MX (100)	MX (100)	MX (100)
Beamline (Wavelength, Å)	DLS I03 (0.9763)	DLS I03 (0.9763)	DLS I03 (0.9763)	DLS I03 (0.9763)	DLS I03 (0.9763)
Detector	EIGER2 XE 16M	EIGER2 XE 16M	EIGER2 XE 16M	EIGER2 XE 16M	EIGER2 XE 16M
Data Processing	Xia2, DIALS	Xia2, DIALS	Xia2, DIALS	Xia2, DIALS	Xia2, DIALS
Space group	$P\ 2_1\ 2_1\ 2_1$	$P\ 2_1\ 2_1\ 2_1$	$P\ 2_1\ 2_1\ 2_1$	$P\ 2_1\ 2_1\ 2_1$	$P\ 2_1\ 2_1\ 2_1$
Cell dimensions					
a,b,c (Å)	49.6, 85.6, 123.3	49.6, 86.0, 123.9	50.0, 88.2, 123.1	50.3, 86.8, 123.3	49.6, 85.9, 123.8
α, β, γ (°)	90, 90, 90	90, 90, 90	90, 90, 90	90, 90, 90	90, 90, 90
No. of molecules/ASU	1	1	1	1	1
No. reflections	42230 (2042)*	39574 (1978)*	43815 (2162)	43435 (2165)*	59190 (2904)*
Resolution (Å)	42.9-1.90 (1.93-1.90)*	50.30-1.95 (1.98-1.95*	50.03-1.90 (1.93-1.90)*	50.28-1.90 (1.93-1.90)*	50.24-1.70 (1.73-1.70)*
R_{merge}	0.103 (2.216)*	0.130 (1.598)*	0.123 (2.296)*	0.102 (1.924)*	0.079 (0.916)*
I/σI	15.10 (1.20)	11.0 (0.80)	12.7 (1.00)*	15.0 (1.4)*	17.3 (1.1)*
CC-half	0.999 (0.592)	0.999 (0.693)	0.999 (0.604)*	0.999 (0.656)*	0.999 (0.824)*
Completeness (%)	100 (99.9)*	100 (100)*	100 (99.4)*	100 (100)*	100 (99.50)*
Multiplicity	13.40 (13.60)*	13.4 (13.60)*	13.4 (12.8)*	13.50 (13.50)*	13.3 (9.9)*
Wilson B value (Å^2)	35.02	33.36	33.73	33.67	24.02
**Refinement**	PHENIX	PHENIX	PHENIX	PHENIX	PHENIX
R_{work}/R_{free+}	0.1821/0.2126	0.1843 /0.2123	0.1909/0.2232	0.1778/0.1991	0.1730/0.1867
No. atoms	3856	3925	3848	3955	4097
- Enzyme	3553	3570	3550	3586	3579
- ligand	62	59	28	38	67
- Water	241	296	270	331	451
Average B-factors	41.41	35.60	43.13	39.89	30.44
- Enzyme	40.82	34.68	43.05	39.41	28.92
- ligand	54.96	50.37	36.88	46.34	45.82
- Water	46.71	43.68	44.94	44.31	40.15
R.m.s deviations					
- Bond lengths (Å)	0.003	0.002	0.003	0.004	0.009
- Bond angles (°)	0.59	0.51	0.59	0.62	1.11

*Highest resolution shell in parentheses. DLS = Diamond Light Source.

Datasets	AspH:Fe:2OG:hFX-Asp (PDB ID: 9FW0)	AspH:Fe:2OG:hFX-Asp (PDB ID: 9HO2)	AspH:Fe:2OG:hFX-(OH)Asp **1.5 s O_2** (PDB ID: 9HO1)	AspH:Fe:2OG:hFX-(OH)Asp (PDB ID: 9HO0)	AspH:Fe:2OG:hFX-(OH)Asp (PDB ID: 9NHZ)
**Data Collection (T in K)**	SFX (298)	SFX (298)	SFX (298)	SFX (298)	SSX (298)
Beamline (Wavelength, Å)	LCLS, MFX (1.50844)	LCLS MFX (1.265982)	LCLS MFX (1.265982)	PAL-XFEL NCI (1.30419)	DLS I24 (0.99998)
Detector	RAYONIX MX340-HS	RAYONIX MX340-HS	RAYONIX MX340-HS	RAYONIX MX225-HS	DECTRIS PILATUS3 6M
Data Processing	cctbx.xfel	cctbx.xfel	cctbx.xfel	cctbx.xfel	DIALS
Space group	$P\ 2_1\ 2_1\ 2_1$	$P\ 2_1\ 2_1\ 2_1$	$P\ 2_1\ 2_1\ 2_1$	$P\ 2_1\ 2_1\ 2_1$	$P\ 2_1\ 2_1\ 2_1$
Number of lattices merged	9379	7561	5940	44386	8736
Cell dimensions					
a,b,c (Å)	50.8, 88.2, 125.2	50.9, 91.0, 125.2	50.9, 90.55, 124.6	50.9, 91.4, 124.1	50.9, 90.4, 124.6
α, β, γ (°)	90, 90, 90	90, 90, 90	90, 90, 90	90, 90, 90	90, 90, 90
No. of molecules/ASU	1	1	1	1	1
No. reflections	41249 (1763)*	51985 (3603)*	49888 (1616)*	32478 (1054)*	22750 (1424)*
Resolution (Å)	26.6-1.95 (1.98-1.95)*	23.57-1.83 (1.86-1.83)*	24.02-1.85 (1.87-1.85)*	39.3-2.14 (2.18-2.14)*	73.18-2.4 (2.46-2.40)*
R_{split}	0.152 (1.904)*	0.170 (0.965)*	0.156 (0.868)*	0.114 (0.641)*	0.203 (1.117)*
I/σI	3.342 (0.414)*	4.675 (0.616)*	5.010 (0.794)*	3.691 (0.758)*	2.826 (0.427)*
CC-half	0.980 (0.165)*	0.970 (0.316)*	0.977 (0.373)*	0.993 (0.498)*	0.968 (0.135)*
Completeness (%)	99.9 (99.9)*	99.9 (99.8)*	99.90 (98.90)*	100 (100)*	98.04 (87.58)*
Multiplicity	46.35 (10.96)*	46.74 (10.37)*	43.92 (10.10)*	280.28 (176.39)*	54.81 (33.52)*
Wilson B value (Å^2)	36.01	33.2	33.87	49.51	55.44
**Refinement**	PHENIX	PHENIX	PHENIX	PHENIX	PHENIX
R_{work}/R_{free+}	0.1763/0.2052	0.1958/0.2209	0.1975/0.2206	0.2081/0.2323	0.2018/0.2371
No. atoms	3682	3720	3672	3544	3516
- Enzyme	3525	3405	3484	3442	3457
- ligand	11	11	19	9	9
- Water	146	179	169	93	50
Average B-factors	44.91	48.37	47.25	60.58	68.14
- Enzyme	44.87	48.34	47.15	60.62	68.32
- ligand	29.92	27.45	29.51	55.17	53.31
- Water	47.10	50.12	51.31	59.60	58.11
R.m.s deviations					
- Bond lengths (Å)	0.008	0.003	0.006	0.007	0.002
- Bond angles (°)	0.92	0.52	0.71	0.57	0.43

*Highest resolution shell in parentheses. SFX = Serial Femtosecond Crystallography. SSX = Serial Synchrotron Crystallography. LCLS = Linac Coherent Light Source. PAL = Pohang Accelerator Laboratory. DLS = Diamond Light Source.

Datasets	AspH:Mn:2OG:hFX-Asn:SCN (PDB ID: 9FVV)	AspH:Fe:2OG:hFX-Asp:NO (PDB ID: 9HO3)
**Data Collection (T in K)**	MX (100)	MX (100)
Beamline (Wavelength, Å)	DLS I03 (0.9763)	DLS I03 (0.9763)
Detector	EIGER2 XE 16M	EIGER2 XE 16M
Data Processing	Xia2, DIALS	AIMLESS
Space group	$P\ 2_1\ 2_1\ 2_1$	$P\ 2_1\ 2_1\ 2_1$
Cell dimensions		
a,b,c (Å)	49.9, 86.1, 123.5	49.8, 86.5, 123.2
α, β, γ (°)	90, 90, 90	90, 90, 90
No. of molecules/ASU	1	1
No. reflections	71102 (3496)*	21768 (3051)*
Resolution (Å)	46.30 - 1.6 (1.63-1.60)*	46.2-2.39 (2.43-2.39)*
R_{merge}	0.061 (1.248)*	0.368 (2.305)*
I/σI	20.60 (2.10)	8.4 (1.0)*
CC-half	1.000 (0.792)	0.994 (0.361)*
Completeness (%)	100 (99.9)*	100 (100)*
Multiplicity	13.20 (12.60)*	12.8 (10.0)*
Wilson B value ($Å^2$)	23.51	47.49
**Refinement**	PHENIX	PHENIX
R_{work}/R_{free+}	0.1666 /0.1909	0.1988/0.2422
No. atoms	4226	3680
- Enzyme	3573	3515
- ligand	26	15
- Water	627	150
Average B-factors	28.36	51.82
- Enzyme	26.56	51.87
- ligand	30.27	49.52
- Water	38.59	50.86
R.m.s deviations		
- Bond lengths (Å)	0.009	0.003
- Bond angles (°)	0.94	0.54

*Highest resolution shell in parentheses. DLS = Diamond Light Source.

Supplementary References

1 Solomon, E. I., Goudarzi, S. & Sutherlin, K. D. O_2 Activation by non-heme iron enzymes. *Biochemistry* **55**, 6363–6374 (2016).

2 Mbughuni, M. M. *et al.* Trapping and spectroscopic characterization of an FeIII-superoxo intermediate from a nonheme mononuclear iron-containing enzyme. *Proc. Natl. Acad. Sci. USA* **107**, 16788–16793 (2010).

3 Price, J. C., Barr, E. W., Glass, T. E., Krebs, C. & Bollinger, J. M. Evidence for hydrogen abstraction from C1 of taurine by the high-spin Fe(IV) intermediate detected during oxygen activation by taurine:α-ketoglutarate dioxygenase (TauD). *J. Am. Chem. Soc.* **125**, 13008–13009 (2003).

4 Krebs, C., Galonić Fujimori, D., Walsh, C. T. & Bollinger, J. M., Jr. Non-heme Fe(IV)–oxo intermediates. *Acc. Chem. Res.* **40**, 484–492 (2007).

5 Solomon, E. I., DeWeese, D. E. & Babicz, J. T., Jr. Mechanisms of O_2 activation by mononuclear non-heme iron enzymes. *Biochemistry* **60**, 3497–3506 (2021).

6 Riggs-Gelasco, P. J. *et al.* EXAFS spectroscopic evidence for an Fe═O Unit in the Fe(IV) intermediate observed during oxygen activation by taurine:α-ketoglutarate dioxygenase. *J. Am. Chem. Soc.* **126**, 8108–8109 (2004).

7 Price, J. C., Barr, E. W., Tirupati, B., Bollinger, J. M. & Krebs, C. The first direct characterization of a high-valent iron intermediate in the reaction of an α-ketoglutarate-dependent dioxygenase: a high-spin Fe(IV) complex in taurine/α-ketoglutarate dioxygenase (TauD) from *Escherichia coli*. *Biochemistry* **42**, 7497–7508 (2003).

8 Srnec, M. *et al.* Electronic structure of the ferryl intermediate in the α-ketoglutarate dependent non-heme iron halogenase SyrB2: contributions to H atom abstraction reactivity. *J. Am. Chem. Soc.* **138**, 5110–5122 (2016).

9 Neidig, M. L. *et al.* Spectroscopic and electronic structure studies of aromatic electrophilic attack and hydrogen-atom abstraction by non-heme iron enzymes. *Proc. Natl. Acad. Sci. USA* **103**, 12966–12973 (2006).

10 C. J. Schofield, R. P. Hausinger, *2-Oxoglutarate-Dependent Oxygenases*, C. J. Schofield, R. P. Hausinger, Eds. (The Royal Society of Chemistry), pp. 1–487 (2015).

11 Pfeffer, I. *et al.* Aspartate/asparagine-β-hydroxylase crystal structures reveal an unexpected epidermal growth factor-like domain substrate disulfide pattern. *Nat. Commun.* **10**, 4910 (2019).

12 Krishnan, A. *et al.* Unusual catalytic strategy by non-heme Fe(II)/2-oxoglutarate-dependent aspartyl hydroxylase AspH. *Chem. Sci.* **15**, 3466–3484 (2024).

13 Elkins, J. M. *et al.* Structure of Factor-inhibiting Hypoxia-inducible Factor (HIF) Reveals Mechanism of Oxidative Modification of HIF-1α. *J. Biol. Chem.* **278**, 1802–1806 (2003).

14 Mitchell, A. J. *et al.* Structural basis for halogenation by iron- and 2-oxo-glutarate-dependent enzyme WelO5. *Nat. Chem. Biol.* **12**, 636–640 (2016).

15 Hou, C. X., Brasnett, A., Rabe, P., Schofield, C. J. & Brewitz, L. Structural and functional consequences of aspartate/asparagine-β-hydroxylase variants causing Traboulsi Syndrome. *J. Biol. Chem.* **302**, 111008 (2026).

16 Brasnett, A. *et al.* Human oxygenase variants employing a single protein FeII ligand are catalytically active. *Angew. Chem. Int. Ed.* **60**, 14657–14663 (2021).

17 McMullen, B. A. *et al.* Complete amino acid sequence of the light chain of human blood coagulation factor X: evidence for identification of residue 63 as β-hydroxyaspartic acid. *Biochemistry* **22**, 2875–2884 (1983).

18 Rabe, P. *et al.* X-ray free-electron laser studies reveal correlated motion during isopenicillin *N* synthase catalysis. *Sci. Adv.* **7**, eabh0250 (2021).

19 Drakenberg, T., Fernlund, P., Roepstorff, P. & Stenflo, J. β-Hydroxyaspartic acid in vitamin K-dependent protein C. *Proc. Natl. Acad. Sci. USA* **80**, 1802–1806 (1983).

20 Stenflo, J. *et al.* Hydroxylation of aspartic acid in domains homologous to the epidermal growth factor precursor is catalyzed by a 2-oxoglutarate-dependent dioxygenase. *Proc. Natl. Acad. Sci. USA* **86**, 444–447 (1989).
21 Srinivas, V. *et al.* High-resolution XFEL structure of the soluble methane monooxygenase hydroxylase complex with its regulatory component at ambient temperature in two oxidation states. *J. Am. Chem. Soc.* **142**, 14249–14266 (2020).
