# Supplementary material for: Structural basis of the promiscuity of the unusual Fe(II) and 2-oxoglutarate dependent human aspartate/asparagine-β-hydroxylase
Source: Nat Commun. 2026 Feb 25;17:4267. doi: 10.1038/s41467-026-69425-w (PMC13168262; doi:10.1038/s41467-026-69425-w)
Supplement: Supplementary file 2 — Reporting Summary [file 41467_2026_69425_MOESM2_ESM.pdf]

nature portfolio

Corresponding author(s): Dr. Lennart Brewitz, Dr. Jan Kern, Prof. Dr. Christopher Schofield, Dr. Patrick Rabe

Last updated by author(s): Dec 20, 2025

Reporting Summary

Nature Portfolio wishes to improve the reproducibility of the work that we publish. This form provides structure for consistency and transparency in reporting. For further information on Nature Portfolio policies, see our Editorial Policies and the Editorial Policy Checklist.

Statistics

For all statistical analyses, confirm that the following items are present in the figure legend, table legend, main text, or Methods section.

n/a	Confirmed	
☐	☒	The exact sample size (*n*) for each experimental group/condition, given as a discrete number and unit of measurement
☐	☒	A statement on whether measurements were taken from distinct samples or whether the same sample was measured repeatedly
☒	☐	The statistical test(s) used AND whether they are one- or two-sided<br>*Only common tests should be described solely by name; describe more complex techniques in the Methods section.*
☐	☒	A description of all covariates tested
☒	☐	A description of any assumptions or corrections, such as tests of normality and adjustment for multiple comparisons
☐	☒	A full description of the statistical parameters including central tendency (e.g. means) or other basic estimates (e.g. regression coefficient) AND variation (e.g. standard deviation) or associated estimates of uncertainty (e.g. confidence intervals)
☒	☐	For null hypothesis testing, the test statistic (e.g. *F*, *t*, *r*) with confidence intervals, effect sizes, degrees of freedom and *P* value noted<br>*Give P values as exact values whenever suitable.*
☒	☐	For Bayesian analysis, information on the choice of priors and Markov chain Monte Carlo settings
☒	☐	For hierarchical and complex designs, identification of the appropriate level for tests and full reporting of outcomes
☒	☐	Estimates of effect sizes (e.g. Cohen's *d*, Pearson's *r*), indicating how they were calculated

Our web collection on statistics for biologists contains articles on many of the points above.

Software and code

Policy information about availability of computer code

Data collection	DIALS ver. 3.24.3, cctbx.xfel,
Data analysis	WinCOOT ver. 0.9.8.7, PHENIX ver 1.21.1.5286, MassLynx 4.2, GraphPad Prism ver. 10.2, Agilent Qualitative Analysis ver. 7.00.7024

For manuscripts utilizing custom algorithms or software that are central to the research but not yet described in published literature, software must be made available to editors and reviewers. We strongly encourage code deposition in a community repository (e.g. GitHub). See the Nature Portfolio guidelines for submitting code & software for further information.

Data

Policy information about availability of data

All manuscripts must include a data availability statement. This statement should provide the following information, where applicable:

- Accession codes, unique identifiers, or web links for publicly available datasets
- A description of any restrictions on data availability
- For clinical datasets or third party data, please ensure that the statement adheres to our policy

All data needed to evaluate the conclusions in the paper are present in the paper and/or the Supplementary Materials. The atomic coordinates and structure factors are deposited in the PDB accession codes: 9FVX (AspH:Fe:2OG:hFx-Asn, MX), 9FVZ (AspH:Fe:2OG:hFx-Asp, MX), 9FVW (AspH:Fe:2OG/succinate:hFx-(OH)Asn, MX), 9FVY (AspH:Fe:2OG/succinate:hFx-(OH)Asp, MX), 9FVU (AspH:Fe:2OG:hFx-Asn:SCN, MX), 9FW0 (AspH:Fe:2OG:hFx-Asp, SFX), 9HO2 (AspH:Fe:2OG:hFX-Asp, SFX), 9HO1 (AspH:Fe:2OG:hFx-(OH)Asp, 1.5 s O2, SFX), 9HO0 (AspH:Fe:2OG: hFx-(OH)Asp, SFX), 9NHZ (AspH:Fe:2OG: hFx-(OH)Asp, SSX), 9FVV (AspH:Mn:2OG:hFx-Asn:SCN, MX) and 9HO3 (AspH:Fe:2OG:hFx-Asp:NO, MX).

Research involving human participants, their data, or biological material

Policy information about studies with human participants or human data. See also policy information about sex, gender (identity/presentation), and sexual orientation and race, ethnicity and racism.

Reporting on sex and gender	NA
Reporting on race, ethnicity, or other socially relevant groupings	NA
Population characteristics	NA
Recruitment	NA
Ethics oversight	NA

Note that full information on the approval of the study protocol must also be provided in the manuscript.

Field-specific reporting

Please select the one below that is the best fit for your research. If you are not sure, read the appropriate sections before making your selection.

☒ Life sciences ☐ Behavioural & social sciences ☐ Ecological, evolutionary & environmental sciences

For a reference copy of the document with all sections, see nature.com/documents/nr-reporting-summary-flat.pdf

Life sciences study design

All studies must disclose on these points even when the disclosure is negative.

Sample size	For crystallographic data, all structures except the time-resolved experiments were collected in at least duplicate; mass spectrometry assays were performed in biological triplicates.
Data exclusions	Obvious outliers (<5%) in mass spectrometry turnover measurements were excluded. For serial crystallography data, a very small fraction of outlier reflections (typically 1–2 per >10,000 images) were removed during standard processing.
Replication	Crystallographic datasets (except time-resolved data) were reproduced independently; mass spectrometry assays were performed in triplicate with consistent results.
Randomization	Randomization was not applicable to this study, as the experiments involve purified proteins and crystallographic assays.
Blinding	Blinding was not applicable, as data collection and analysis relied on automated crystallographic and spectrometric pipelines.

Reporting for specific materials, systems and methods

We require information from authors about some types of materials, experimental systems and methods used in many studies. Here, indicate whether each material, system or method listed is relevant to your study. If you are not sure if a list item applies to your research, read the appropriate section before selecting a response.

Materials & experimental systems

n/a	Involved in the study	
☒	☐	Antibodies
☒	☐	Eukaryotic cell lines
☒	☐	Palaeontology and archaeology
☒	☐	Animals and other organisms
☒	☐	Clinical data
☒	☐	Dual use research of concern
☒	☐	Plants

Methods

n/a	Involved in the study	
☒	☐	ChIP-seq
☒	☐	Flow cytometry
☒	☐	MRI-based neuroimaging

Plants

Seed stocks	NA
Novel plant genotypes	NA
Authentication	NA
